# Topology-preserving coronary vessel segmentation in X-ray angiography using ResNet50-MASAG-UNet

**DOI:** 10.3389/fradi.2026.1949450

**Published:** 2026-10-14

**Authors:** G. Sunilkumar, P. Kumaresan

**Affiliations:** 1 School of Computer Science Engineering and Information Systems (SCORE), Vellore Institute of Technology, Vellore, India; 2 Centre For Artificial Intelligence Research (C-FAIR), Vellore Institute of Technology, Vellore, India

**Keywords:** attention gates, coronary artery disease, ResNet50-MASAG-UNet, vessel segmentation, vessel visualization, X-ray coronary angiography

## Abstract

Coronary artery disease occurs when the accumulation of fatty atheroma deposits narrows the coronary blood vessels, thereby reducing blood flow to the myocardium. To identify these narrowings, cardiologists use X-ray coronary angiography (XCA). However, these angiographic images are often blurry and noisy, making vessels difficult to view clearly. To enhance visibility, we proposed an image improvement technique that combines three filtering approaches: Bilateral filtering, unsharp masking, and Contrast Limited Adaptive Histogram Equalization (CLAHE), to remove noise, sharpen vessel edges, and improve brightness balance, producing clearer vessel images to support subsequent analysis. Our proposed ResNet50-MASAG-UNet integrates a Multi-Scale Adaptive Spatial Attention Gate (MASAG) at each skip connection of a ResNet50-UNet, enabling the decoder to attend selectively to coronary vessel regions while suppressing non-vascular activations from catheters and bones. A topology-aware loss is used to preserve vessel connectivity by maintaining centreline continuity. Together, these elements enable more accurate and complete coronary vessel segmentation. The proposed model achieves improved segmentation performance on XCA images, attaining a Dice coefficient of 88.3%, a Jaccard Index of 79.1%, a precision of 86.4%, and a recall of 90.5%. It outperforms the standard UNet baseline and provides clearer vessel segmentation, demonstrating improved potential for coronary vessel analysis.

## 1 Introduction

Coronary artery disease (CAD) is a non-communicable disease in which fatty atheroma forms inside the cardiac arteries, narrowing the blood vessels and reducing blood flow to the heart. This ailment increases the risk of cardiac attacks. CAD is one of the leading causes of death globally, affecting millions of individuals and causing nearly 17.9 million mortalities every year. The aetiology of coronary artery disease is multifaceted, including modifiable lifestyle factors, environmental influences, and genetic predispositions. Common risk factors for CAD include diabetes, hypertension, tobacco use, hyperlipidaemia, obesity, chronic renal disease, dietary habits, family predisposition, homocystinuria, and psychological stress (1, 2). X-ray coronary angiography (XCA) is the standard approach for diagnosing CAD, detecting stenosis, quantifying its severity, and supporting real-time decision-making (3). Other medical imaging techniques such as ultrasound, single-photon emission computed tomography, and magnetic resonance angiography (MRA) are less invasive but do not provide the detailed XCA imaging. Different angiographic approaches exist for different vascular regions of the body, but cardiologists rely on coronary angiography to evaluate the arteries and veins of the heart (4).

Stenotic lesion evaluation is crucial for determining the severity of stenosis (5). Quantitative coronary angiography measures the diameter of the coronary arteries (6) and the percentage of occlusion (7) through a structured luminal analysis. Using a catheter and contrast dye, XCA produces detailed vessel images for accurate diagnosis (8). XCA generates image sequences that require segmentation techniques, such as minimization of energy, machine learning, and path searching, but often lack temporal coherence between frames, which can lead to imaging errors, such as missing vessels (9, 10).

Accurate segmentation of coronary vessels from XCA images is still a challenging problem. The vessels are thin, low contrast, and vary across patients and imaging conditions. They are also often hidden by structures such as catheters and heart shadows. As a result, standard models may miss small vessel branches that are useful for clinical analysis. This work is motivated by three specific limitations of current XCA segmentation. First, datasets such as DCA1 are small, which causes overfitting when deep encoders are trained from scratch. Second, standard skip connections pass background noise from catheters, bones, and heart shadows directly to the decoder, weakening thin-vessel recovery. Third, pixel-wise losses such as binary cross-entropy (BCE) and Dice optimize region overlap but do not preserve vessel connectivity, producing fragmented centrelines. To address these issues, we developed a ResNet50-MASAG-UNet model for accurate segmentation of coronary arteries. The developed framework combines bilateral filtering, unsharp masking, and Contrast Limited Adaptive Histogram Equalization (CLAHE) to improve vessel visibility, enhance contrast, and sharpen the edges of coronary vessels. Additionally, the model extracts vessels while suppressing interference from catheters, bones, and overlapping structures, resulting in enhanced vessel continuity and more complete vessel segmentation.

This article presents the following research contributions:

- We developed a new vessel segmentation algorithm for the visibility and contrast of vessels that integrates bilateral filtering, unsharp masking, and CLAHE to reduce noise, enhance vessel boundaries, and improve contrast in XCA images for better vascular visibility.
- We used a ResNet50 encoder part to extract important features such as edges and vessel shapes, helping to improve performance even with the limited DCA1 dataset.
- We adapted the Multi-Scale Adaptive Spatial Attention Gate (MASAG) to replace the standard skip connections, so that encoder and decoder features jointly focus on coronary-vessel regions while suppressing background noise.
- The proposed model reduces broken coronary vessel predictions and improves segmentation quality by merging BCE, Dice, and clDice loss components.
- We evaluated the performance metrics Dice Coefficient, Accuracy, Jaccard Index, Precision, Recall, and Specificity compared with state-of-the-art models.

This article is structured as follows: Section 2 provides an overview of existing work related to our proposed methodology. Section 3 outlines the architectural steps for data preparation, the techniques used for preprocessing, and the proposed method. Section 4 outlines the experimental framework, performance criteria, segmentation outcomes, and comparisons with state-of-the-art models. Section 5 examines the results and reflects on the proposed methodology. Section 6 concludes with the principal contributions of the proposed model and future enhancements.

## 2 Related work

Coronary artery segmentation from XCA is challenging because of the intricate vascular anatomy, overlapping vessels, and contrast variation between vessels and background, all of which make it difficult to separate vessels accurately (11). Early approaches used filter-based and region-growing methods to address these challenges (12). The coronary vasculature is commonly categorized into four types: normal vessels, occluded vessels, stenotic arteries, and vessels with reduced perfusion (13), but manual categorization is laborious and expensive. Despite advances in AI and digital image processing for segmenting and classifying coronary vasculature, accurately differentiating vessels from the background remains difficult.

Convolutional neural networks (CNNs) have been widely adopted for automated angiographic segmentation. Notable CNN-based approaches include UNet and its variants for biomedical image segmentation (14, 15), as well as specialized architectures designed to learn effectively from limited angiographic data (16, 17). The latter proposed a UNet-based model for segmenting left and right coronary arteries, though it was limited to specific vessels. Other studies have explored multi-stage CNN frameworks for artery localization and refinement, as well as vessel segmentation models capable of distinguishing vessels from artefacts in real-time imaging scenarios (18).

Preprocessing techniques that enhance image contrast are essential for improving the visibility of blood vessels relative to surrounding tissues, thereby enabling accurate segmentation. CLAHE enhances local intensity contrast and is particularly effective in regions with non-uniform illumination (19). Unsharp masking improves edge definition by subtracting a blurred version of the image from the original, thereby enhancing fine structural details (20). Histogram equalization redistributes pixel intensity values to improve overall contrast and highlight structural details (21). The top-hat transform employs morphological operations to separate bright structures, such as blood vessels, from the background, thereby enhancing vascular visibility (22). Bilateral filtering preserves important edge details and reduces noise, ensuring that coronary vessel boundaries remain sharp and are not blurred (23).

Attention mechanisms, including CBAM (24) and attention gates (25), help models concentrate on important image regions. However, these approaches often provide limited interaction between encoder and decoder vessel features. To address this issue, the Multi-Scale Adaptive Spatial Attention Gate (MASAG) (26) enables more detailed attention through two-way communication between these features across spatial and channel information.

Recent studies on coronary angiography segmentation have moved beyond simple pixel-level detection to focus on vessel continuity, accurate labelling, complex vessel shape, and the recovery of thin distal vessels. DSSNet uses two parallel paths, one capturing overall vessel information and the other focusing on edges, fused to preserve thin vessel boundaries in low-contrast images (27). UCNet combines a GAN with an improved UNet to segment vessels and identify coronary segments, performing well even with limited training data (28). M^2^ CA-Net applies a 2D DCT-based multi-frequency channel attention block to combine spatial and frequency information to better capture fine vessel details. HR-UMamba++ uses rotation-aligned multi-directional scanning with a high-resolution bypass and a dense UNet++ decoder to preserve thin distal branches (34). While these recent methods advance vessel continuity and feature extraction using frequency attention, sequence-based scanning, or generative models, they do not employ two-way, multi-scale attention at the skip connections. Our work differs by adapting the MASAG module to XCA within a ResNet50-UNet and pairing it with a topology-aware BCE + Dice + clDice loss and an XCA-specific preprocessing pipeline to improve the segmentation of thin, low-contrast coronary vessels while preserving connectivity.

Another group of studies improves segmentation by explicitly constraining vessel topology and morphology. Snake-RWKV maintains vessel continuity when 2D images are converted to 1D sequences, using an adaptive scanning and shifting mechanism to follow curved vessels (35). TOFF-Net introduces a vascular connectivity preservation loss and an irrelevant-vessel-removal module to maintain connectivity and suppress false-positive structures (36). LIS-Net couples a frequency-guided dual-decoder with a coronary morphology loss that combines region- and boundary-based information to reduce fragmentation in semi-supervised learning (37). AMU-Net adds a MambaASPP module and a centreline-boundary Dice term to improve small-vessel connectivity and topological continuity (38). MRA-DPNet models geometric continuity with a direction-aware asymmetric convolution and a differentiable vessel-width consistency loss for anatomically realistic vessel widths (39). Together, these studies show that maintaining vessel connectivity, topology, and morphology—not pixel-level accuracy alone—is essential for clinically reliable coronary segmentation.

However, on this XCA dataset, a topology-preserving loss such as clDice has not, to our knowledge, been combined with a two-way, multi-scale skip-attention module. This work addresses that gap by integrating a topology-aware hybrid loss with the MASAG-based framework to improve structural accuracy in coronary vessel segmentation.

## 3 Proposed architecture

We present the proposed reliable coronary artery segmentation methodology, an end-to-end pipeline consisting of three main phases, as shown in Figure 1. First, the acquired dataset is pre-processed using techniques such as bilateral filtering, unsharp masking, and dual CLAHE to improve vessel quality, enhance contrast, and suppress noise from XCA images. Next, the segmentation process is carried out using the ResNet-50-MASAG-UNet architecture, which is designed to segment coronary vessels efficiently. Finally, the robustness of the model is verified using a topology-aware hybrid loss function and performance metrics, and we explain how the design manages complex backgrounds and overlapping structures to achieve efficient coronary artery segmentation.

**Figure 1:**
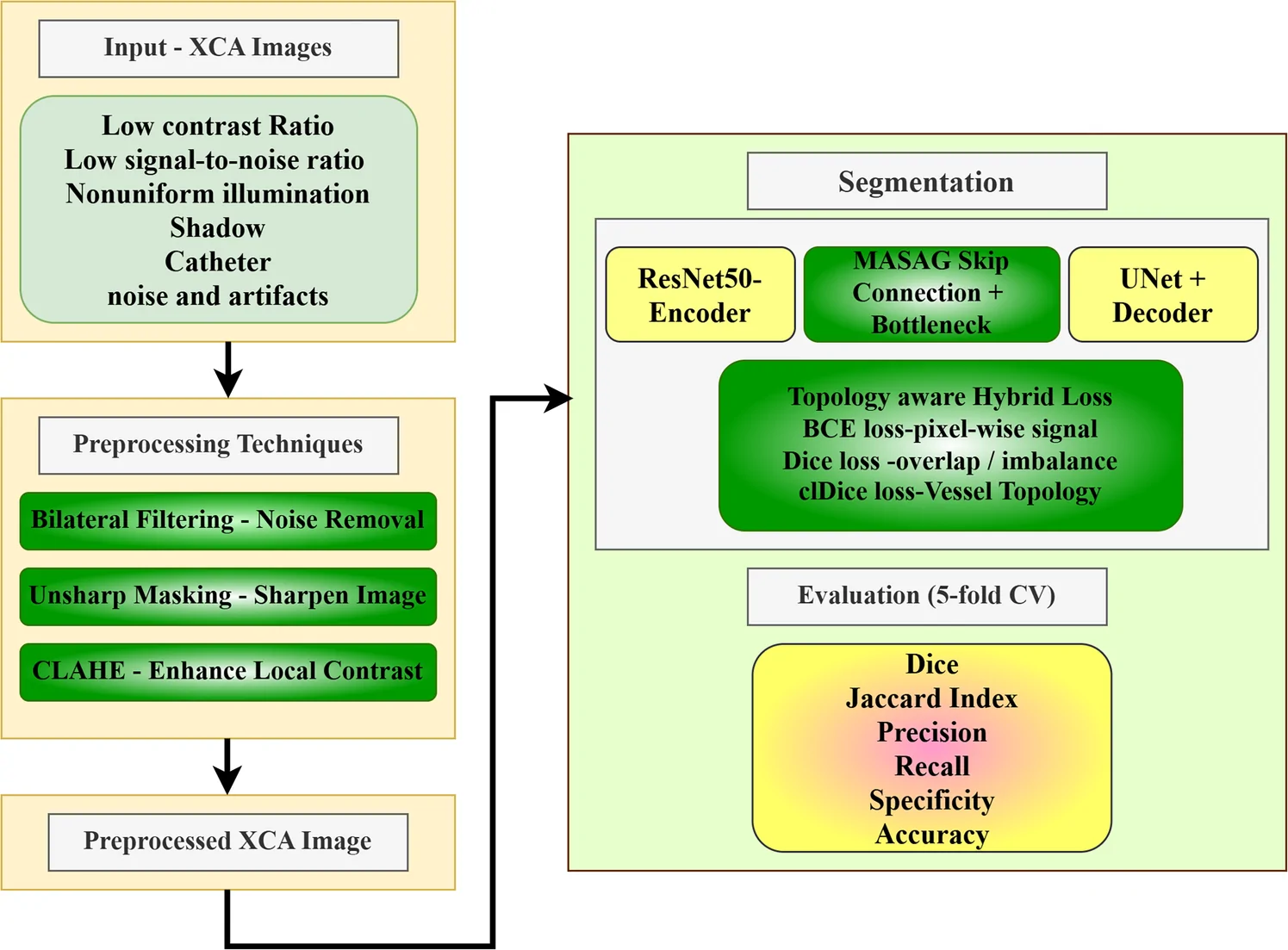
Proposed ResNet50-MASAG-UNet workflow diagram.

### 3.1 Preparation of dataset

The proposed model was evaluated on the publicly available DCA1 database of X-ray coronary angiograms (40), accessed from Mendeley http://doi.org/10.17632/4wvf54td8d.1. The dataset contains 134 grayscale XCA images of size 300 × 300 pixels, each with a ground-truth vessel mask prepared by expert cardiologists, as shown in Figure 2. The images were originally collected at the Cardiology Department of the Mexican Social Security Institute (IMSS). A five-fold cross-validation strategy was used for reliable evaluation: in each fold, the images were split into training and validation sets. Because 134 is not divisible by five, four folds used 107 training and 27 validation images, and one fold used 108 training and 26 validation images. The public DCA1 database is distributed as individual angiogram images without patient identifiers, so cross-validation was therefore performed at the image level. Because patient labels are not available, patient-level independence between the training and validation splits cannot be verified; this is a limitation of the dataset. As DCA1 provides a single still angiogram image per case (not video sequences), repeated frames from the same collection do not appear across folds.

**Figure 2:**
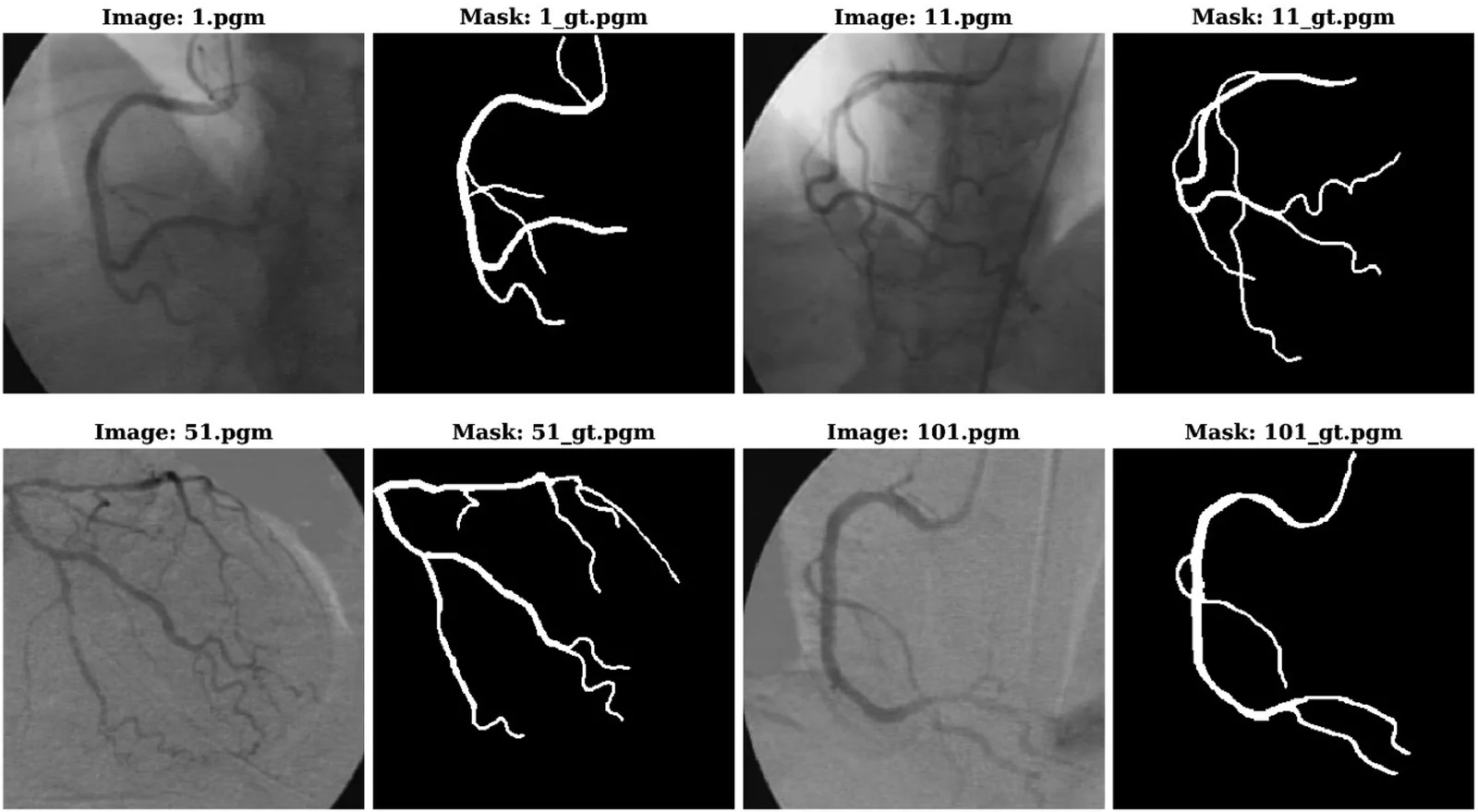
Raw XCA image with annotated mask images.

Angiogram analysis and segmentation are critical due to low XCA image contrast, lack of proper brightening, poor signal-to-noise ratio, and disturbances from nearby structures, as illustrated in Figure 3. These problems reduce image visibility and make accurate vessel detection difficult.

**Figure 3:**
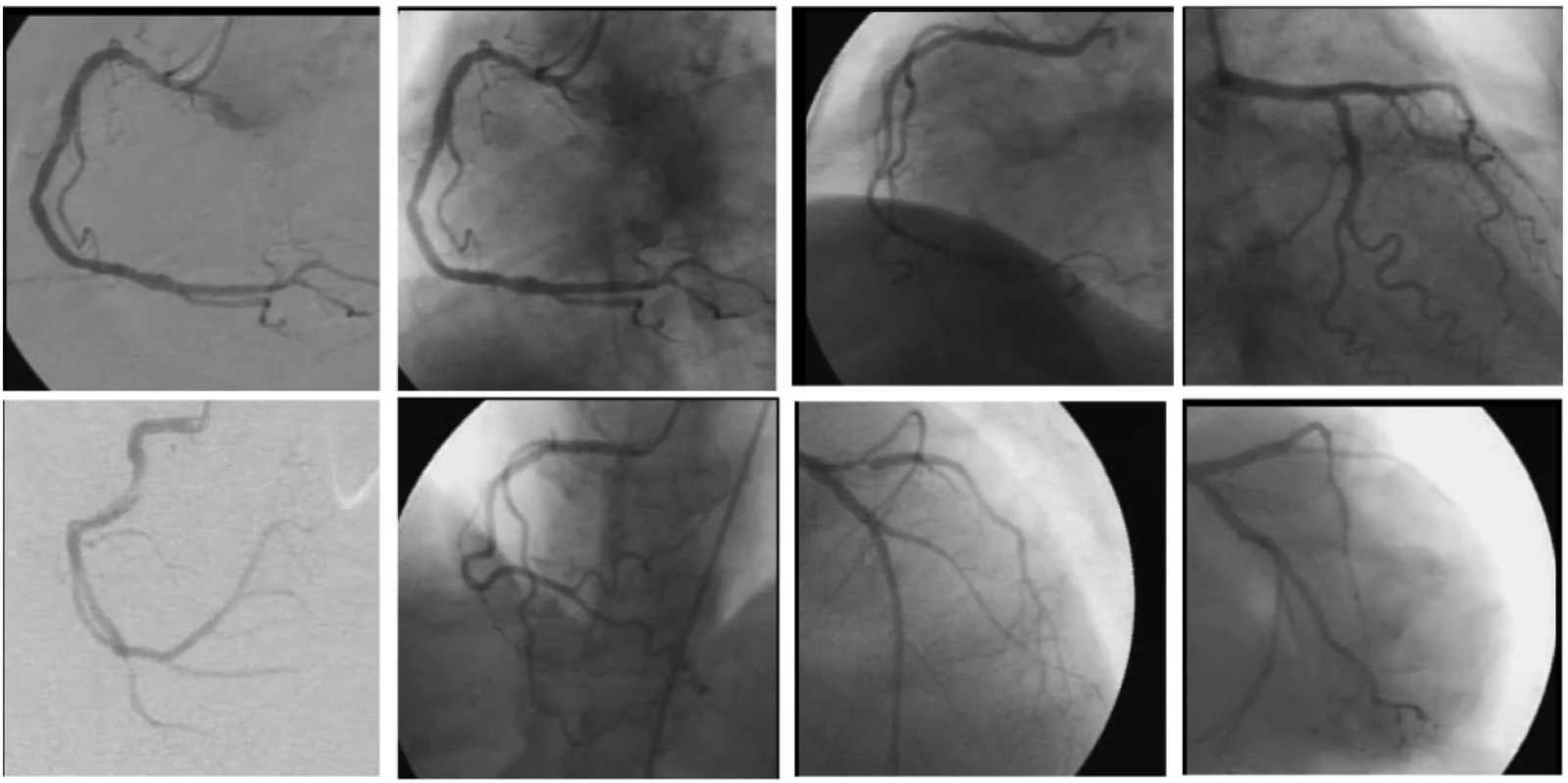
X-ray angiography samples (uneven illumination, low contrast ratios, and high noise, catheter, shadow).

### 3.2 Image visualization and vessel enhancement

To ensure efficient coronary vessel segmentation, a combined preprocessing pipeline is utilized to enhance the raw XCA image quality. This procedure enhances image contrast, reduces noise, and reveals important regions in vessels. It also addresses common problems in XCA images, including low contrast, uneven brightness, and overlapping structures that obscure vessel boundaries. The preprocessing workflow includes three main techniques, as shown in Figure 4. First, bilateral filtering (23) with a neighbourhood diameter *d* = 9, *σ*_color = 75, *σ*_space = 75 targets the low signal-to-noise ratio by smoothing background noise while preserving vessel edges. Next, unsharp masking (20), with a Gaussian kernel of 9 × 9, *σ* = 10.0, and an amount factor *α* = 1.5, sharpens fine spatial details and clarifies tiny vascular structures. Finally, CLAHE (19) with a clip limit of 3.0 and an 8 × 8 tile grid, improves local vessel-background contrast and corrects uneven brightness. The moderate clip limit of 3.0 limits noise amplification in low-intensity regions, while the 8 × 8 grid provides local adaptation to the uneven illumination typical of XCA images without introducing block artefacts.

**Figure 4:**
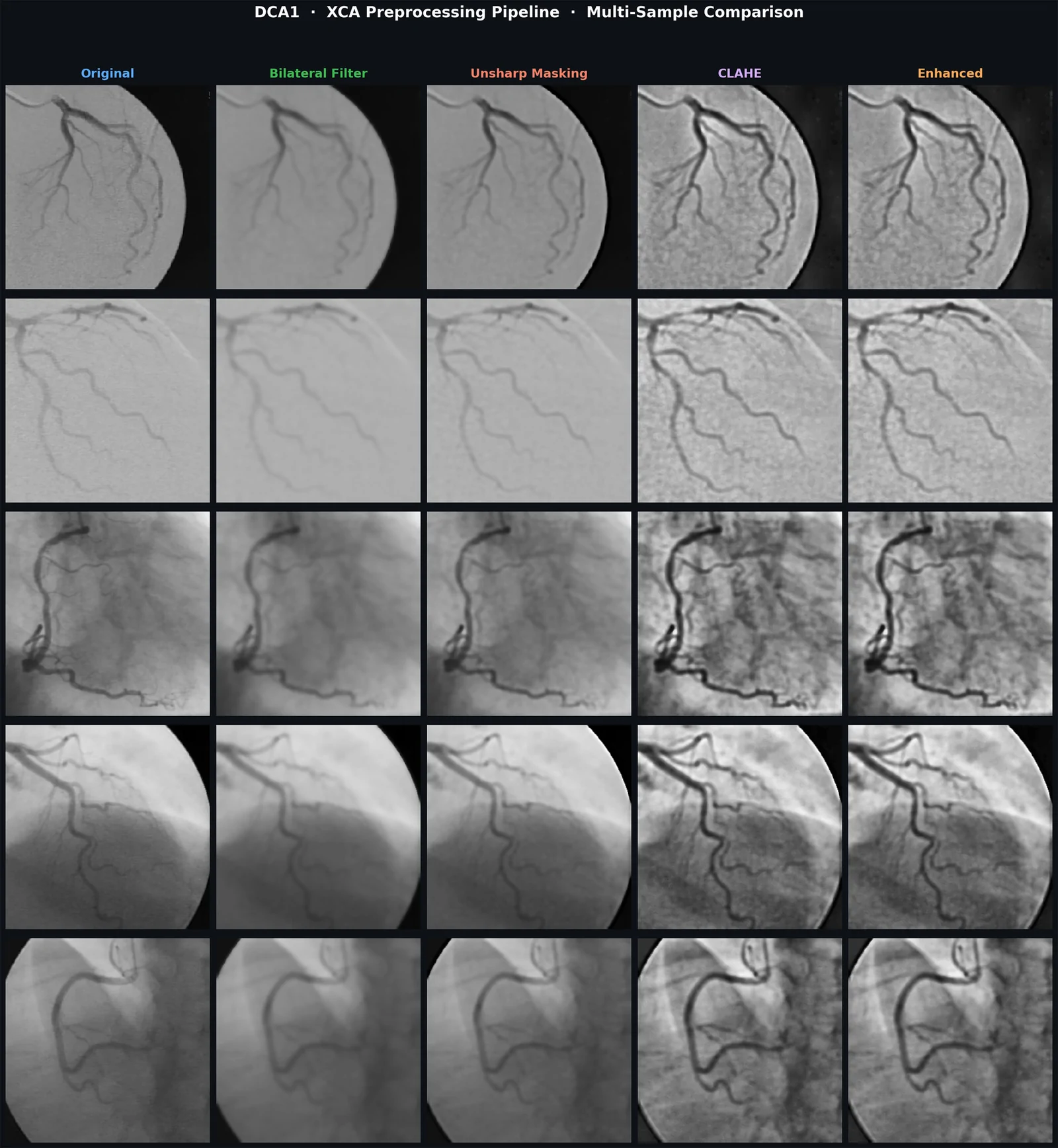
Raw XCA image with pre-processed images.

The pre-processing parameters for bilateral filtering, unsharp masking, and CLAHE were set to commonly used values and kept the same for all images to ensure consistent pre-processing. Sample XCA images were visually checked to confirm that the settings improved vessel contrast without increasing noise.

The pre-processing steps were applied in the following order: bilateral filtering, followed by unsharp masking and CLAHE. Bilateral filtering is applied first to reduce noise while preserving vessel edges. Unsharp masking is then used to sharpen the vessel edges. Finally, CLAHE is applied to improve local contrast. This order helps enhance the vessels while avoiding unnecessary amplification of noise.

As shown in the comparative views in Figure 4, the combination of bilateral filtering, unsharp masking, and CLAHE transforms the raw XCA input. These complementary techniques work together to suppress noise, enhance vessel edges, and improve interior contrast. As a result, the improved coronary vessels become easier for accurate segmentation.

### 3.3 ResNet50-MASAG-UNet: a hybrid approach to coronary angiography segmentation

To improve coronary vessel segmentation in XCA, we proposed an enhanced UNet architecture designed for XCA segmentation, as shown in Figure 5. The model incorporates two important enhancements. First, a ResNet50 encoder is applied to extract important features from the XCA images, aiding in capturing both tiny vessel structures and broader background information. Second, MASAG-based skip connections are added to improve the network's focus on important vessel regions while reducing the effects of noise and overlapping non-vessel anatomy. These enhanced multi-scale feature maps are then passed to the decoder, allowing the model to provide accurate reconstructions of the coronary vessel tree and support subsequent vessel analysis. Although DCA1 images are grayscale, the single intensity channel is replicated three times to create a three-channel (256 × 256 × 3) input, enabling the use of ImageNet-pretrained ResNet50 encoder weights directly, followed by standard ImageNet normalization, which is then applied using the standard per-channel mean (0.485, 0.456, 0.406) and standard deviation (0.229, 0.224, 0.225). By facilitating stable gradient propagation and learning deep XCA image details, the ResNet50 encoder helps improve the overall efficiency of the segmentation process. ResNet50's residual connections make training more effective and assist in capturing vessel details more efficiently. In the standard UNet, skip connections directly pass encoder features to the decoder, which can introduce unwanted background information such as catheters or bone shadows. To address this, MASAG modules are added to each skip connection from Conv1 to Conv4. Each MASAG takes encoder features as the signal input and the corresponding decoder features as the guidance**.**

**Figure 5:**
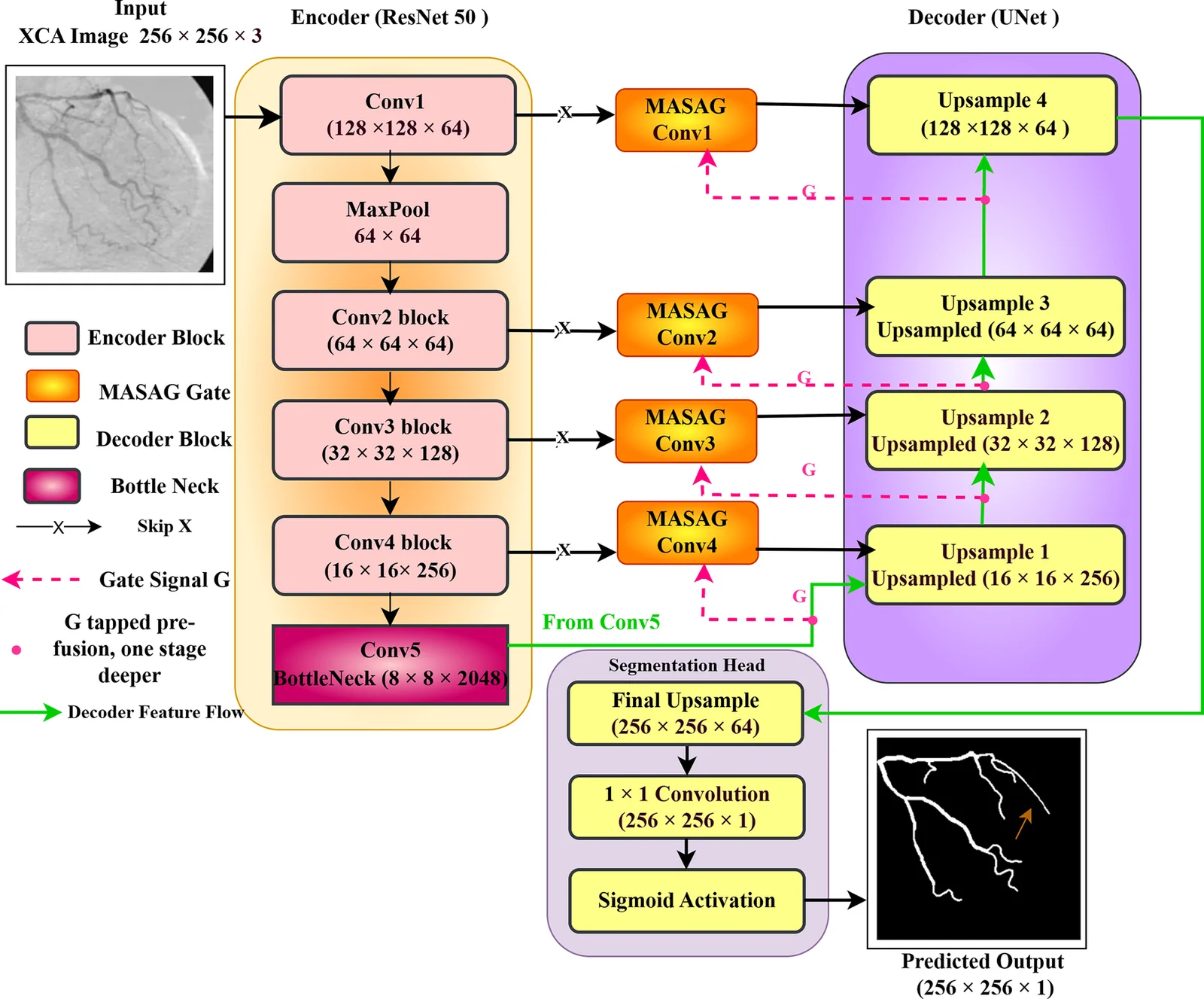
Proposed ResNet50-MASAG-UNet framework for XCA image segmentation.

The symbols and tensor dimensions used in the following equations are summarized in Table 1.

**Table 1:** Notation of symbols and tensor dimensions used in the proposed method.

Symbol	Meaning	Dimension
X	Encoder skip feature (signal input to MASAG)	H × W × C
G	Decoder guidance feature (in MASAG, Section 3.4)	H × W × C
F_fused	Fused multi-scale feature (Equation 1)	H × W × C
S	Spatial selection map (Equation 2)	H × W × 2
w_X, w_G	Spatial attention weights for encoder/decoder paths (Equation 3)	H × W × C
X′, G′	Reweighted encoder/decoder features (Equation 3)	H × W × C
X′′ G′′	Cross-modulated features (Equation 4)	H × W × C
F_inter	Interaction feature (Equation 5)	H × W × C
*α*	Channel attention vector (Equation 6)	H × W × C
X_out	Recalibrated MASAG output (Equation 7)	H × W × C
C	Channels per skip level	64,64,128,256 (Conv1—Conv4)
P	Predicted segmentation (metrics/loss)	256 × 256
G	Ground-truth mask (metrics/loss)	256 × 256
S_P, S_G	Predicted/ground-truth soft-skeletons (Equations 9, 10)	256 × 256
ʘ	Element-wise multiplication	–
⊕	Element-wise addition	–
*Σ*	Sigmoid function	–
*Ε*	Smoothing constant (1 × 10^−7^)	Scalar

### 3.4 Multi-scale adaptive spatial attention gate internal design

The MASAG module follows the four-stage design of Kolahi et al. (26).: multi-scale feature extraction, spatial selection, cross-modulation, and recalibration. It refines each encoder skip feature X using the decoder guidance signal G (Here, G denotes the decoder guidance feature; in the loss and evaluation sections, G is reused to denote the ground-truth mask.) In this work, the internal operations from Equations 1–7 are used as originally proposed. Our adaptations are at the integration level. Unlike the original work, which uses a transformer encoder, the module is integrated into a ResNet50 encoder and UNet decoder architecture, applied at the four encoder skip connections, and its per-level input channel dimensions are matched to the ResNet50 skip connections listed in Table 3 (*C* = 64,64,128,256 for Conv1-Conv4). Because the encoder skip feature X and the decoder guidance G originate at different resolutions, G is aligned to the spatial size of X before fusion via global average pooling and upsampling, shown as the Up[GAP(G)] term in Equation 1, and 1 × 1 convolutions project features to the common channel dimension C.

#### 3.4.1 Multi-scale feature extraction

The encoder feature X *_is_* processed through two parallel paths:

1. A depth-wise separable conv 3 × 3 to capture fine vessel details,
2. A dilated convolution rate = 2 to capture wider vessel structures such as curves and branches.

The outputs are added, and the global decoder context from G is added to form the fused feature in Equation 1.

$$F_{\mathrm{fused}}=\;\mathrm{ReLU}(\mathrm{BN}(\mathrm{DSCon}v_{3\;\times \;3}\;(X)))⊕\mathrm{ReLU}(\mathrm{BN}(\mathrm{DilCon}v_{3\;\times \;3,\;}\;r=2\;(X)))⊕\mathrm{Up}(\mathrm{GAP}(G))$$ (1)

where $\mathrm{DSCon}v_{3\;\times \;3}$ denotes the depth-wise separable 3 × 3 convolution, $\mathrm{DilCon}v_{3\;\times \;3,\;}\;r=2$ is the dilated convolution with rate 2, BN is batch normalization, ReLU is the rectified linear unit, GAP is global average pooling, Up is upsampling, G is the decoder guidance feature, and ⊕ is element-wise addition.

#### 3.4.2 Spatial selection

The fused feature is projected to two channels and passed through a Softmax to produce a spatial selection map, which generates two attention weights as shown in Equations 2, 3:

$$S=\mathrm{softmax}(\mathrm{BN}(\mathrm{Con}v_{1\;\times \;1}(F_{\mathrm{fused}})))$$ (2)

The Softmax in Equation 2 is applied along the channel axis, i.e., across the two output channels at each spatial location, so that the attention weights sum to one per pixel and form a spatial selection between the encoder and decoder feature paths.

$$w\_X=\;\sigma \;(\mathrm{Con}v_{1\;\times \;1}\;\;\hat{X}(S)),\;w\_G=\sigma (\mathrm{Con}v_{1\;\times \;1}\hat{G}(S))\;X^{\prime }=X⊙\;w\_X,G^{\prime }=G⊙w\_G$$ (3)

Two separate 1 × 1 convolutions with sigmoid activation are applied to the selection map S to produce the spatial attention weights w_X and w_Gε R ^(H × W × C) for the encoder and decoder paths, respectively. The encoder and decoder features are then reweighted as X′ = X ʘ w_X, G′ = G ʘ w_G.

#### 3.4.3 Cross modulation

The weighted encoder and decoder features modulate each other to enable two-way interaction by using Equations 4, 5:

$$X^{\prime \prime }=X^{\prime }⊙\mathrm{ReLU}\;(\mathrm{Con}v_{1\;\times \;1}(G^{\prime })),\;G^{\prime \prime }=G^{\prime }⊙\mathrm{ReLU}(\mathrm{Con}v_{1\;\times \;1}(X^{\prime }))$$ (4)

$$F_{\mathrm{inter}=X^{\prime \prime }⊕G^{\prime \prime }}$$ (5)

#### 3.4.4 Recalibration

A channel attention vector *α* is computed and applied to X to produce the refined output, as shown in Equations 6, 7:

$$\alpha =\sigma (\mathrm{Con}v_{1\;\times \;1}(\mathrm{ReLU}(\mathrm{Con}v_{1\;\times \;1}(F_{\mathrm{inter}}))))$$ (6)

$$X_{\mathrm{out}}=X⊙\alpha$$ (7)

where ʘ is element-wise multiplication, ⊕ is addition, GAP is global average pooling, BN is batch normalization, and σ is the sigmoid function. The refined $X_{\mathrm{out}}$ is passed to the decoder.

The multi-level operation of MASAG across four skip connection levels is illustrated in Figure 6: at Stage 1, it captures fine details such as thin and small vessel branches, preserving edge information. Stage 2 enhances vessel boundary clarity, improving the definition of vessel walls. At Stage 3, MASAG reduces background noise, such as cardiac tissue, helping to better separate vessels from surrounding structures. At the deepest Stage 4, it captures the overall structure of the coronary artery tree, providing global context. These refined features from all levels are then passed to the decoder, resulting in more accurate, continuous vessel centrelines and reduced background noise in vessel segmentation compared to standard methods.

**Figure 6:**
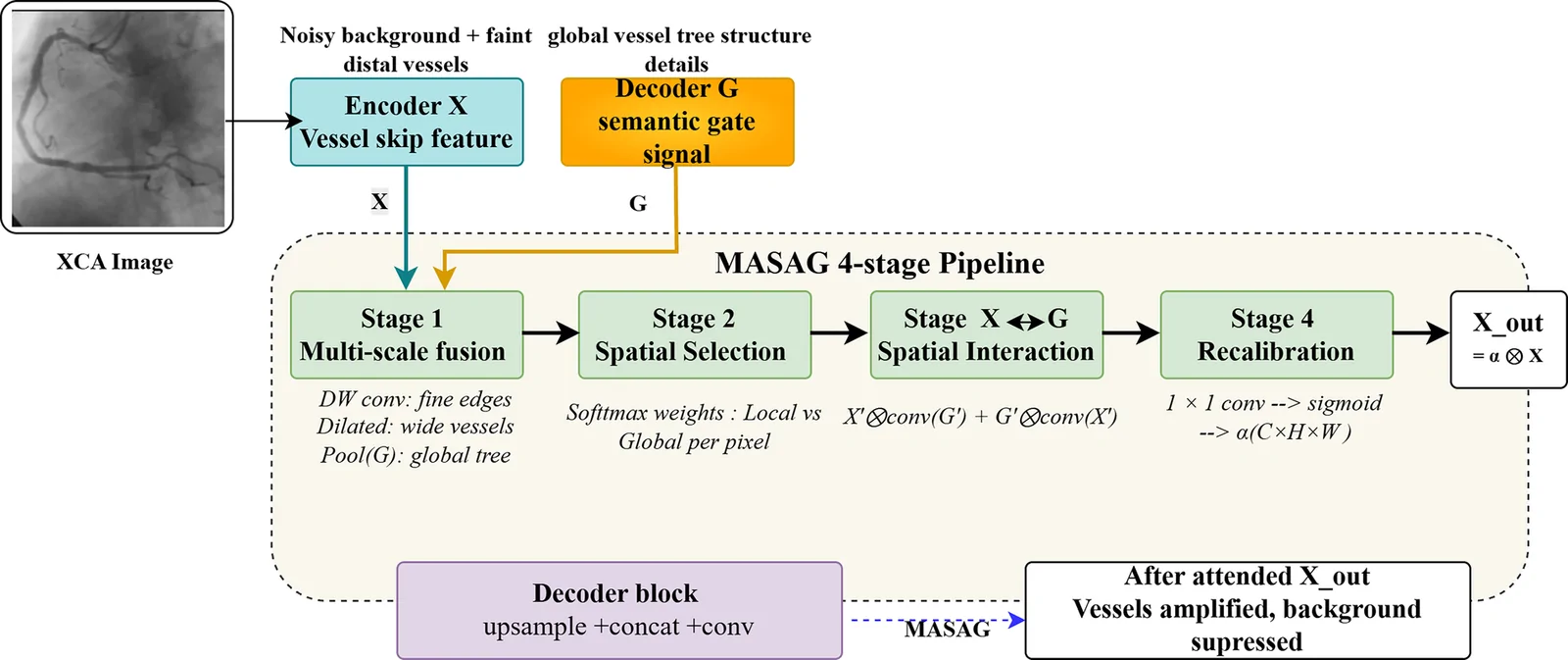
Architecture of the proposed multi-scale adaptive spatial attention gate (MASAG).

Table 2 shows the layer-by-layer details of the MASAG module, where C is the number of input channels for the encoder skip connection. In Stage 1, multi-scale features are combined using depth-wise and dilated 3 × 3 convolutions along with global decoder information. In Stage 2, spatial selection weights are generated using 1 × 1 convolution followed by Softmax. In Stage 3, encoder and decoder features interact through cross-modulation. In Stage 4, the features are refined using channel attention with a reduction ratio of 8.

**Table 2:** Layer-by-layer specification of the MASAG module.

Stage	Operation	Kernel/rate	Output channels	Activation
1	Depth-wise Conv + BN	3 × 3	C	ReLU
1	Dilated Conv + BN	3 × 3, rate 2	C	ReLU
1	Global path: GAP(G) + Upsample	–	C	–
1	Fusion (Add) → F_fused	–	C	–
2	Conv + BN (selection)	1 × 1	2	Softmax
2	Two 1 × 1 Convs → w_X, w_G	1 × 1	C	Sigmoid
3	Cross-Conv on X′, G′,	1 × 1	C	ReLU
3	Modulate → F_inter	–	C	–
4	Reduce	1 × 1	C/8	ReLU
4	Attention α	1 × 1	C	Sigmoid
4	Output:X_out = Xʘα	–	C	–

**Table 3:** Encoder skip connection locations and channel dimensions for each MASAG module.

MASAG	Encoder layer	Feature size (H × W)	Channels (C)
Conv1	Conv1_relu	128 × 128	64
Conv2	Conv2_block3_1_relu	64 × 64	64
Conv3	Conv3_block4_1_relu	32 × 32	128
Conv4	Conv4_ block6_1_relu	16 × 16	256

Each MASAG module is applied to one encoder skip connection, taking the encoder feature as input X and the corresponding decoder feature as guidance G. Table 3 lists the encoder layer, feature size, and channel dimension (C) at each of the four skip locations Conv1-Conv4. For a MASAG at a given skip level with input shape H × W × C, the tensors X, G, F_fused, X′, G′, and X_out_ all have shape H × W × C, while the spatial selection map S has shape H × W × 2.

### 3.5 Topology-aware hybrid loss function

BCE and Dice focus on pixel overlap, so they may still generate discontinuous vessel predictions even when the overall area is correct. To address this, clDice is used to preserve vessel continuity by comparing the centreline structures.

The clDice metric can be calculated using Equation 8.

$$\mathrm{clDice}(P,G)=\frac{2(T_{\mathrm{Prec}}.T_{\mathrm{sens}})}{T_{\mathrm{Prec}}+T_{\mathrm{sens}}}$$ (8)

Here, P denotes the predicted segmentation and G the ground-truth mask. (Note: G here refers to the ground truth, distinct from the decoder guidance feature G used in Section 3.4.)

Here, $T_{\mathrm{Prec}}$ and $T_{\mathrm{sens}}$ are evaluated using centreline matching, as shown in Equations 9, 10:

$$T_{\mathrm{Prec}}=\frac{S_{P}.G+\varepsilon }{\Sigma S_{P\;}+\varepsilon }$$ (9)

$$T_{\mathrm{sens}}=\frac{S_{G}.P+\varepsilon }{\Sigma S_{G\;}+\varepsilon }$$ (10)

This metric verifies whether P, which denotes the predicted segmentation, covers G, the ground truth mask.

Here, $S_{P}$ and $S_{G}$ denote the predicted and ground-truth skeletons, respectively. The skeletons $S_{P}$ and $S_{G}\;$ are obtained using a differentiable soft-skeletonization operation, in which iterative min-and-max pooling approximate morphological erosion and dilation, producing skeletons that remain differentiable for gradient-based training (44). A small smoothing constant *ε* = 1 × 10 ^−^ ^7^ is added to the numerator and denominator of $T_{\mathrm{Prec}}$ and $T_{\mathrm{sens}}$ to ensure numerical stability and prevent division by zero. When the coronary vessels are broken, the resulting skeleton becomes fragmented, which reduces both $T_{\mathrm{Prec}}$ and $\;T_{\mathrm{sens}}$, raising the loss value.

Since clDice and Dice are similarity metrics (higher is better), the corresponding losses are defined as their complements: L_Dice = 1-Dice, L_clDice = -clDice (P, G), while L_BCE is the standard binary cross-entropy. In the results and tables, Dice and clDice refer to these metrics, whereas L_Dice, L_clDice, and L_BCE denote the training losses.

Finally, Equation 11 combines pixel-level and anatomical consistency into a single hybrid loss function.

$$L_{\mathrm{total}}=\frac{1}{3}\;L_{\mathrm{BCE}}+\;\frac{1}{3}\;L_{\mathrm{Dice}}+\frac{1}{3}\;L_{\mathrm{clDice}}$$ (11)

Equal weights (1/3 each) are used because the three terms address complementary objectives such as pixel-level accuracy (BCE), region overlap (Dice), and topological connectivity (clDice) and are on a comparable numerical scale, so no single term dominates the gradient. A loss-weight sensitivity analysis in Table 11 confirms that performance is stable across a range of clDice weights and peaks at the equal-weight (balanced) setting, supporting this choice.

The hybrid loss balances three contributions: $L_{\mathrm{BCE}}$ refines internal pixels, $L_{\mathrm{Dice}}$ manages class imbalance, and $L_{\mathrm{clDice}}$ enforces topological vessel connectivity. Together, these terms support accurate segmentation of coronary vessels.

The clDice loss uses the differentiable soft-skeletonization of Shit et al. (44), which iteratively applies soft morphological erosion and dilation (via min/max pooling with a 3 × 3 kernel) to approximate the vessel centreline. We used five iterations, which is sufficient to extract the thin coronary vessel skeletons at the 256 × 256 resolution.

#### 3.5.1 Hausdorff Distance95 (HD95)

HD95 captures boundary-level segmentation accuracy using Equation 12, penalizing large local deviations between predicted and ground truth vessel boundaries at the 95th percentile. It is computed as the 95th percentile of the symmetric boundary distances between the predicted and ground-truth masks, on the 256 × 256 masks (resized from the original 300 × 300 images), and is reported in pixels. No empty predictions occurred in our experiments, so the distance was defined in all cases.

$$HD_{95}\;(A,B)=\max\{HD_{95}(d(a\to B)),HD_{95}(d(b\to A))\}$$ (12)

where HD95 is the 95th percentile Hausdorff distance, P is the predicted boundary, and G is the ground-truth boundary.

#### 3.5.2 Betti number error $\left(\beta_{0}\right)$

The Betti-0 error measures the topological correctness of the predicted vessel structure using Equation 13. A lower value indicates fewer broken vessel segments and spurious connections relative to the ground truth. Connected components are identified using 8-connectivity, which is appropriate for thin, diagonally oriented coronary vessels.

$$\mathrm{Bett}i_{0}\;\mathrm{Error}=|B_{0}\;(P)-B_{0}\;(G)|$$ (13)

*B*_0_(*P*) is the number of connected components in the prediction and $B_{0}\;(G)$ is the number in the ground truth.

### 3.6 Two-phase training schedule

#### 3.6.1 Phase 1: encoder frozen

The ResNet50 backbone remains fixed while only the MASAG gates and decoder layers are trained for 25 epochs, using a learning rate of 1 × 10 ^−^ ^4.^ This stage allows the decoder and attention modules to learn stable features without changing the pretrained encoder weights.

#### 3.6.2 Phase 2: full fine-tuning

The entire ResNet50 encoder is then unfrozen and fine-tuned together with the MASAG gates and decoder for the remaining 25 epochs, using the same learning rate of 1 × 10 ^−^ ^4^, allowing all layers to adapt to the XCA domain.

The two phases therefore total 50 epochs, and the checkpoint with the best validation Dice is selected as the final model. (R1:MinorC3).

## 4 Experimental design and performance analysis

### 4.1 Experimental configuration and implementation details

The proposed segmentation framework was developed using the Keras–TensorFlow framework with Python 3.10.13 and trained in the Kaggle environment. All experiments were performed using an NVIDIA Tesla P100 GPU with 16GB RAM and a Xeon CPU. The ResNet50 encoder was initialized with pretrained ImageNet weights to leverage transfer learning, while the decoder and MASAG modules were randomly initialized. The encoder was then fine-tuned using the two-phase schedule described in Section 3.6. On this hardware, training required approximately 4 min, and the average inference time was approximately 22 ms per image, indicating the model is practical for use.

For preprocessing, different image resolutions were tested to analyse the effect on segmentation efficiency. CLAHE, with a clip limit of 3.0 and a tile grid size of 8 × 8, was applied to enhance vessel visibility and suppress noise in the background. A five-fold cross-validation strategy was used on all 134 DCA1 XCA images. The final performance values are reported as mean ± standard deviation across five folds, providing a more reliable evaluation compared to a single train–validation split.

DCA1 XCA images were used to train the ResNet50-MASAG-UNet model for vessel segmentation. Input images were normalized to the range [0,1] and resized to 256 × 256 pixels. Input images were resized using bilinear interpolation, while the ground-truth masks were resized using nearest-neighbour interpolation to preserve the binary vessel labels and avoid degrading thin vessels. No data augmentation was applied; the models were trained on the original preprocessed images within each cross-validation fold. A threshold of 0.5 was used to binarize the sigmoid output, as it is the standard decision boundary for a probability output and requires no dataset-specific tuning, thus avoiding an additional hyperparameter. To address class imbalance, the BCE, Dice, and clDice losses were combined into the topology-aware hybrid loss used for training. The model was trained for 50 epochs with a batch size of 8 using the Adam optimizer (*β*_1_ = 0.9, *β*_2_ = 0.999) and a fixed learning rate of 1 × 10^−4^. For each fold, the checkpoint with the best validation performance (highest validation Dice) was selected as the final model, and the reported metrics correspond to these checkpoints. The two-phase fine-tuning schedule (Section 3.6) controls which layers are trained in each phase; the learning rate was kept the same (1 × 10^−4^) in both phases, and no learning-rate scheduler was used. Model performance was then evaluated using the Dice coefficient and related metrics. The proposed ResNet50-MASAG-UNet has 37,397,633 parameters in total (37,340,529 trainable and 57,104 non-trainable). At an input resolution of 256 × 256, the model requires approximately 32.5 GFLOPs per forward pass. Training required approximately 4 min per fold on the NVIDIA Tesla P100 GPU, and the average inference time was approximately 22 ms per image. Peak GPU memory usage was approximately 4.2 GB during training (batch size 8) on the 16 GB Tesla P100 GPU.

To ensure a fair comparison, all baseline models of UNet and UNet++ were trained using the same settings as the proposed model: identical preprocessing steps such as bilateral filtering, unsharp masking, and CLAHE; image size of 256 × 256; normalization; Adam optimizer; learning rate 1 × 10^−4^; batch size of 8; and 50 training epochs, along with the same five-fold cross-validation strategy on the DCA1 dataset. Binary cross-entropy loss was used for these baselines, as they do not use the topology-based hybrid loss. The two-phase training strategy was used only for the proposed ResNet50-MASAG-UNet.

### 4.2 Evaluation measures

Since vessel pixels occupy only a small area of XCA images, accuracy alone cannot properly reflect segmentation performance. The Dice coefficient provides a better evaluation by measuring vessel similarity with the ground truth. Jaccard gives a strict overlap check, while precision and specificity ensure non-vessel areas are not wrongly detected. Recall is important because a missed vessel could affect subsequent analysis. Because vessel pixels are far outnumbered by background, accuracy and specificity are the least informative metrics here; we therefore emphasize Dice, recall, clDice, and HD95 to evaluate the proposed ResNet50-MASAG-UNet, as denoted in Equations (14)–(19).

$$\mathrm{Accuracy}=\frac{\mathrm{TP}+\mathrm{TN}}{\mathrm{TP}+\mathrm{TN}+\mathrm{FP}+\mathrm{FN}}$$ (14)

$$\mathrm{Dice}=\;\frac{2\times |P\cap G|\;}{\left|P|+|G\right|}$$ (15)

$$\mathrm{IoU}=\;\frac{\left|P\cap \;G\right|}{\left|P\;\cup \;G\right|}$$ (16)

$$\mathrm{Precision}=\frac{\mathrm{TP}\;}{\mathrm{TP}+\mathrm{FP}}$$ (17)

$$\mathrm{Recall}\;=\frac{\mathrm{TP}\;}{\mathrm{TP}+\mathrm{FN}}$$ (18)

$$\mathrm{Specificity}\;=\;\frac{\mathrm{TN}\;}{\mathrm{TN}+\mathrm{FP}}$$ (19)

### 4.3 Segmentation outcomes

A comprehensive ablation study was conducted to evaluate the contributions of both the preprocessing pipeline and architectural design, as each preprocessing step gradually improves performance in segmentation across various models.

Table 4 shows the effect of adding each preprocessing step to the ResNet50-MASAG-UNet. Performance improves steadily from the raw input of 80.2% Dice to the full pipeline with bilateral filtering, unsharp masking, and CLAHE, achieving 88.3% Dice, confirming that each step contributes to better vessel segmentation.

**Table 4:** Preprocessing ablation on ResNet50-MASAG-UNet: individual techniques (BF, UM, CLAHE), pair-wise combinations, and the full pipeline on the DCA1 dataset.

Model	Dice (%)	Jaccard (%)	Precision (%)	Recall (%)	Accuracy (%)	Specificity (%)	clDice metric (%)	HD95 (px)	Betti
ResNet50-MASAG-UNet (Raw)	80.2 ± 1.18	67.4 ± 1.32	79.1 ± 1.38	82.4 ± 1.52	94.8 ± 0.18	96.2 ± 0.28	78.8 ± 1.30	12.6 ± 1.45	4.8 ± 0.62
ResNet50-MASAG-UNet + BF (Only)	81.6 ± 1.12	68.8 ± 1.25	80.2 ± 1.31	83.4 ± 1.43	95.1 ± 0.16	96.4 ± 0.25	80.0 ± 1.23	11.2 ± 1.35	4.2 ± 0.58
ResNet50-MASAG-UNet + UM (Only)	81.0 ± 1.15	68.1 ± 1.28	79.8 ± 1.35	82.8 ± 1.48	94.9 ± 0.17	96.3 ± 0.26	79.5 ± 1.25	11.8 ± 1.40	4.5 ± 0.60
ResNet50-MASAG-UNet + CLAHE (Only)	83.8 ± 0.98	71.9 ± 1.12	82.4 ± 1.20	85.6 ± 1.38	95.6 ± 0.14	97.0 ± 0.20	82.3 ± 1.10	8.6 ± 1.10	3.2 ± 0.50
ResNet50-MASAG-UNet + BF + UM	85.4 ± 0.94	74.5 ± 1.08	83.9 ± 1.18	87.6 ± 1.38	96.2 ± 0.12	97.4 ± 0.18	84.2 ± 1.02	7.1 ± 0.88	2.8 ± 0.44
ResNet50-MASAG-UNet + BF + CLAHE	86.8 ± 0.82	76.2 ± 0.95	84.9 ± 1.15	88.9 ± 1.25	96.6 ± 0.10	97.7 ± 0.16	86.5 ± 0.90	5.8 ± 0.65	2.2 ± 0.35
ResNet50-MASAG-UNet + UM + CLAHE	86.1 ± 0.85	75.4 ± 0.98	84.3 ± 1.18	88.2 ± 1.30	96.4 ± 0.11	97.5 ± 0.17	85.8 ± 0.92	6.4 ± 0.72	2.3 ± 0.33
ResNet50-MASAG-UNet + BF + UM + CLAHE	88.3 ± 0.72	79.1 ± 0.85	86.4 ± 1.12	90.5 ± 1.34	97.1 ± 0.08	98.2 ± 0.14	89.1 ± 0.81	4.1 ± 0.30	1.5 ± 0.26

As shown in Table 5, in this ablation, all configurations were trained under the same single-phase schedule, with only MASAG (on/off) and the loss function varied; the two-phase fine-tuning was applied only to the final proposed model. The baseline ResNet50-UNet with BCE loss attains a Dice coefficient of 74.8% and an IoU of 60.8%. When the combined Dice + BCE loss is used, the effectiveness increases to 76.1% Dice and 62.0% IoU. The use of clDice further improves the results to 77.3% Dice and 63.8% IoU, showing the benefit of topology-aware learning. After integrating MASAG into the architecture, a clear improvement is observed. The ResNet50-MASAG-UNet with BCE loss reaches 83.1% Dice and 72.4% Jaccard Index, showing the effectiveness of attention-based feature refinement. Further gains are attained with the Dice + BCE loss, producing 85.9% Dice and 75.8% IoU, while training with the clDice loss gives the best Dice metric of 87.1% and 77.5% IoU. The full model, ResNet50-MASAG-UNet + Dice + BCE + clDice, achieves the best results with a Dice coefficient of 88.3% and IoU of 79.1%, along with a higher precision of 86.4% and recall of 90.5%. These results demonstrate that combining multiple complementary loss functions with MASAG leads to consistent performance gains.

**Table 5:** Ablation study of architectural and loss-function components on the DCA1 dataset.

Model	Dice (%)	Jaccard (%)	Precision (%)	Recall (%)	Accuracy (%)	Specificity (%)	clDice (%)	HD95 (px)	Betti
ResNet50-UNet + BCE	74.8 ± 1.12	60.8 ± 1.24	73.5 ± 1.45	76.2 ± 1.56	92.6 ± 0.18	94.8 ± 0.32	72.4 ± 1.38	19.2 ± 1.94	7.1 ± 0.82
ResNet50-UNet + Dice + BCE	76.1 ± 1.05	62.0 ± 1.18	78.3 ± 1.38	74.5 ± 1.48	93.1 ± 0.15	95.8 ± 0.28	74.8 ± 1.20	14.3 ± 1.42	5.2 ± 0.58
ResNet50-UNet + clDice	77.3 ± 1.02	63.8 ± 1.14	79.1 ± 1.32	75.8 ± 1.44	93.5 ± 0.14	96.1 ± 0.26	78.6 ± 1.15	11.8 ± 1.28	4.1 ± 0.52
ResNet50-MASAG-UNet + BCE	83.1 ± 0.94	72.4 ± 1.05	81.6 ± 1.22	85.0 ± 1.38	95.4 ± 0.12	96.9 ± 0.22	81.9 ± 1.02	9.6 ± 0.94	3.8 ± 0.48
ResNet50-MASAG-UNet + Dice + BCE	85.9 ± 0.86	75.8 ± 0.96	84.2 ± 1.18	87.9 ± 1.36	96.2 ± 0.10	97.4 ± 0.18	84.7 ± 0.94	7.4 ± 0.68	3.1 ± 0.42
ResNet50-MASAG-UNet + clDice	87.1 ± 0.78	77.5 ± 0.90	85.3 ± 1.14	89.2 ± 1.35	96.7 ± 0.09	97.8 ± 0.16	86.3 ± 0.88	6.2 ± 0.55	2.3 ± 0.38
ResNet50-MASAG-UNet + Dice + BCE + clDice	88.3 ± 0.72	79.1 ± 0.85	86.4 ± 1.12	90.5 ± 1.34	97.1 ± 0.08	98.2 ± 0.14	89.1 ± 0.81	4.1 ± 0.30	1.5 ± 0.26

In the architectural ablation, removing MASAG from the ResNet50 leads to a clear drop in performance, with Dice decreasing by about 8-10% and the Jaccard Index by a similar margin. This highlights the importance of attention-guided skip connections. Overall, the proposed ResNet50-MASAG-UNet achieves the best results, and qualitative analysis shows that MASAG effectively reduces false positives in non-vessel regions such as catheters and bones.

To evaluate how many skip connections benefit from MASAG, we varied the number of MASAG-equipped skips from one to four in Table 6. Performance improves steadily with each added module, and using MASAG at all four skips yields the best result, with a Dice score of 88.3%.

**Table 6:** Ablation of the number of MASAG-equipped skip connections on the DCA1.

Model	Dice (%)	Jaccard (%)	Precision (%)	Recall (%)	Accuracy (%)	Specificity (%)	clDice (%)	HD95(px)	Betti
ResNet-MASAG-UNet (1 Skip-Bottleneck)	83.2 ± 0.95	71.2 ± 1.10	82.0 ± 1.25	85.5 ± 1.40	95.5 ± 0.14	96.8 ± 0.22	83.5 ± 1.10	8.2 ± 0.95	3.4 ± 0.48
ResNet-MASAG-UNet (2 Skips)	85.1 ± 0.88	74.0 ± 1.02	83.8 ± 1.18	87.4 ± 1.32	96.1 ± 0.12	97.3 ± 0.18	85.4 ± 1.01	6.5 ± 0.78	2.6 ± 0.40
ResNet-MASAG-UNet (3 Skips)	86.9 ± 0.80	76.8 ± 0.92	85.1 ± 1.12	89.0 ± 1.25	96.6 ± 0.10	97.8 ± 0.15	87.3 ± 0.90	5.2 ± 0.55	2.0 ± 0.32
ResNet-MASAG-UNet (4 Skips—Full)	88.3 ± 0.72	79.1 ± 0.85	86.4 ± 1.12	90.5 ± 1.34	97.1 ± 0.08	98.2 ± 0.14	89.1 ± 0.81	4.1 ± 0.30	1.5 ± 0.26

Figure 7 shows the training and validation curves for a representative fold, selected as the fold whose final validation Dice was closest to the mean across all five folds. Training accuracy increases from approximately 85% to nearly 100%, while validation accuracy improves from about 84% to 97%–98% over 50 epochs, with both losses decreasing steadily. Specifically, training loss reduces from 0.90 to nearly 0.01, and validation loss declines from approximately 0.88 to around 0.02. The close alignment of training and validation curves indicates stable optimization on the available dataset; however, due to the limited dataset size, broader generalization cannot be conclusively established and is addressed through five-fold cross-validation.

**Figure 7:**
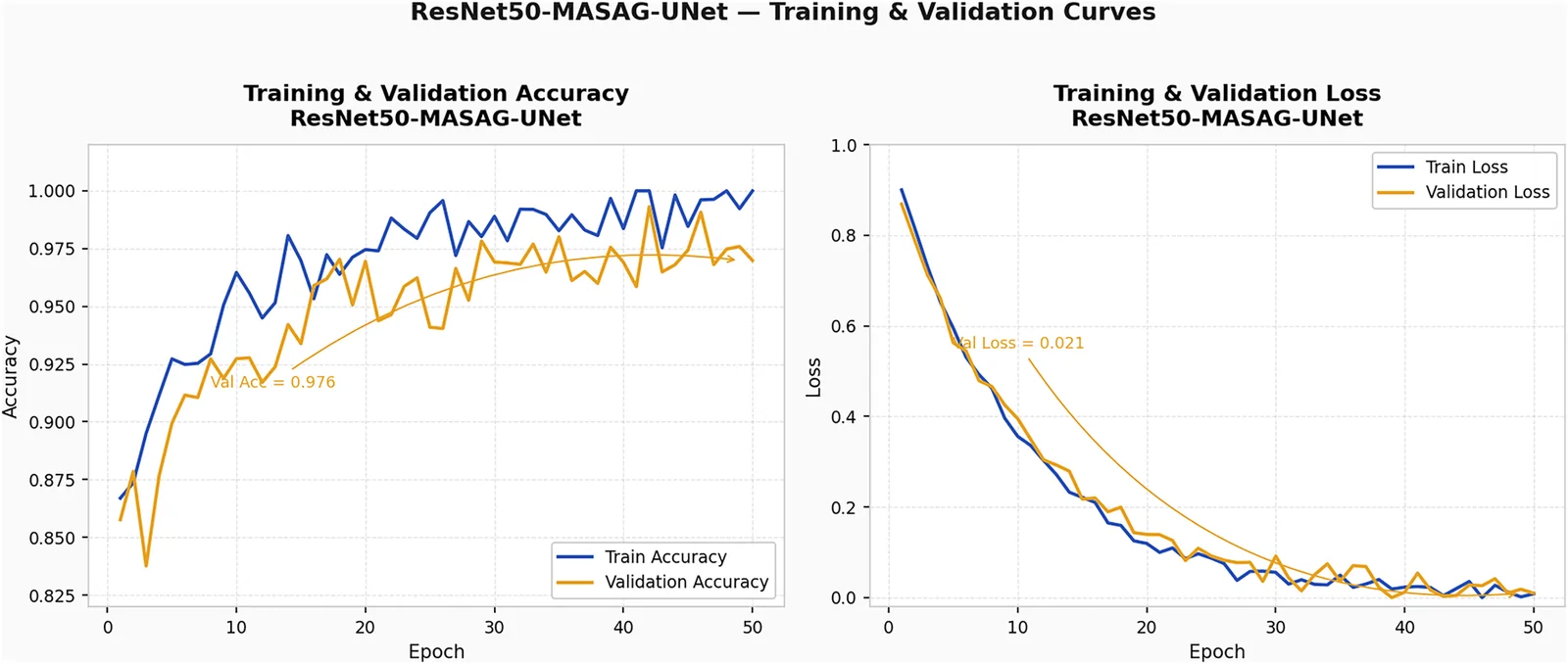
Training and validation accuracy and loss curves of the proposed ResNet50-MASAG-UNet model.

Figure 8 shows the average learning curves across all five folds. The training and validation losses decrease and stabilize, while validation Dice improves from about 35% to 88%. The small shaded regions show low variation between folds, indicating stable training and good generalization without major overfitting.

**Figure 8:**
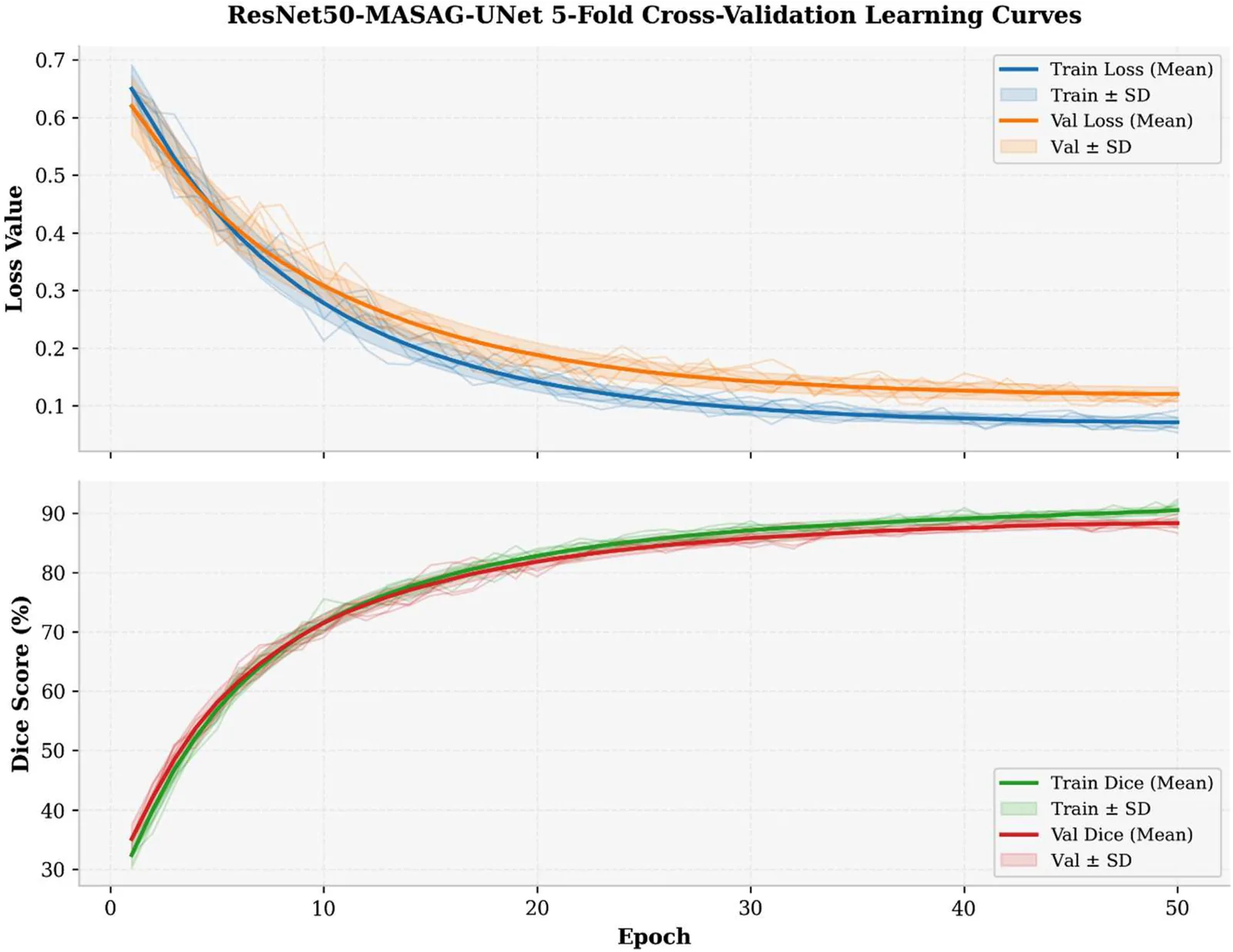
Aggregated fivefold cross-validation learning curves for ResNet50-MASAG-UNet. Top: training and validation loss; bottom: training and validation Dice score. Solid lines show the mean across the five folds, shaded regions ± one standard deviation, and light lines show individual folds.

Figure 9 represents the clDice results across the five folds. The validation clDice increases to about 88%–90% and closely follows the training curve. The narrow ± SD bands indicate stable and consistent vessel connectivity across all folds.

**Figure 9:**
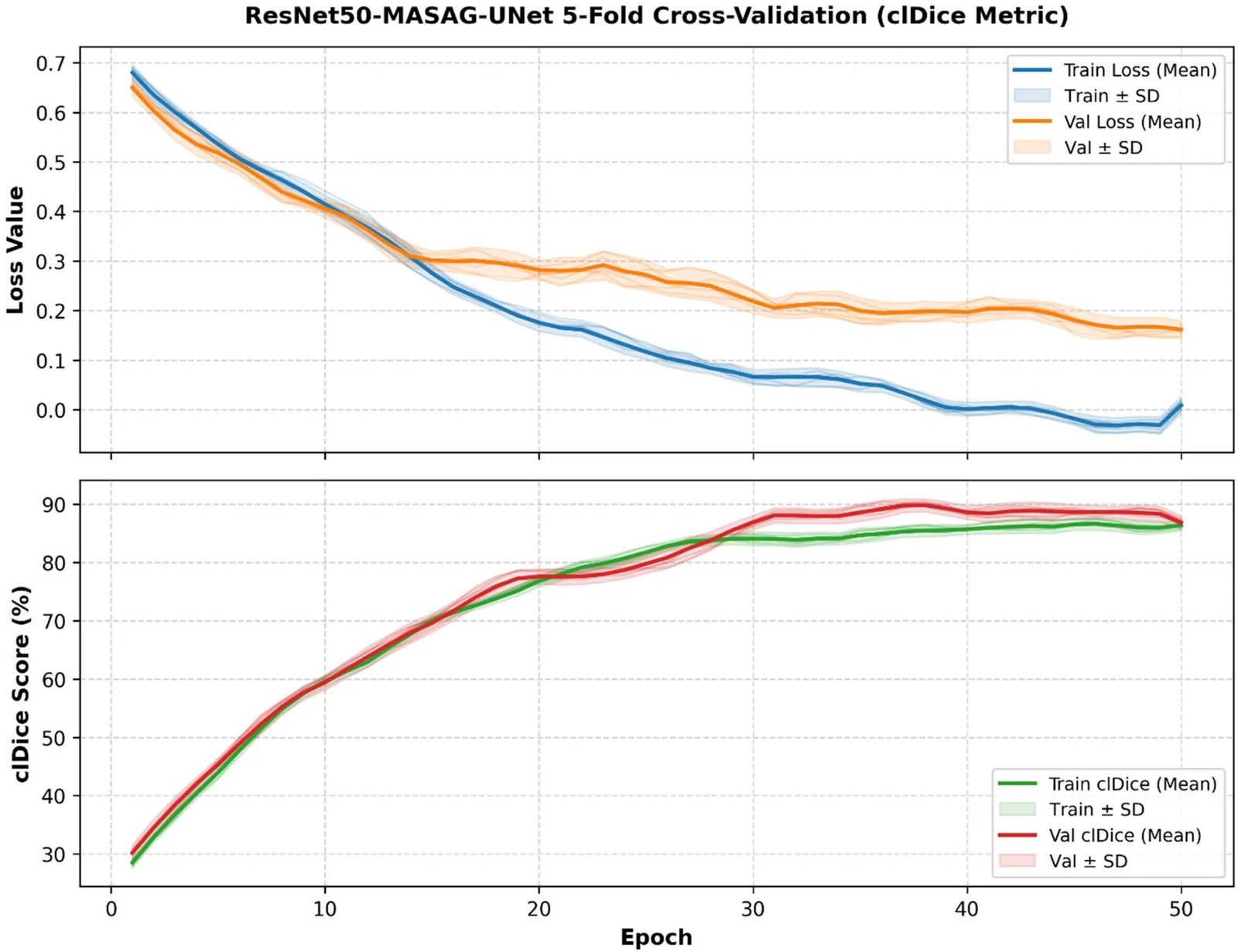
Aggregated five-fold cross-validation curves for the clDice metric (ResNet50-MASAG-UNet). Top: training and validation loss; bottom: training and validation clDice score. Solid lines show the mean across the five folds; shaded regions show ± one standard deviation.

### 4.4 Performance comparison against state-of-the-art methods

This section compares the proposed ResNet50-MASAG-UNet with baseline architectures and with recently reported methods on the DCA1 dataset.

Table 7 presents the comparison of the proposed model with baseline architectures (UNet, UNet++, and ResNet50-UNet without Attention) on the DCA1 dataset under matched training settings. The two-phase fine-tuning was applied only to the proposed model.

**Table 7:** Comparison of the proposed model with baseline architectures on the DCA1 dataset.

Model	Dice (%)	Jaccard (%)	Precision (%)	Recall (%)	Accuracy (%)	Specificity (%)	clDice (%)	HD95 (px)	Betti
UNet	74.0 ± 1.25	59.0 ± 1.30	76.2 ± 1.42	72.8 ± 1.55	92.4 ± 0.20	95.1 ± 0.35	71.2 ± 1.40	18.6 ± 1.82	6.8 ± 0.75
UNet + +	74.7 ± 1.18	58.0 ± 1.28	75.8 ± 1.38	72.4 ± 1.48	92.2 ± 0.18	94.9 ± 0.32	72.1 ± 1.35	17.9 ± 1.64	6.5 ± 0.68
ResNet50-UNet (No Attention)	76.1 ± 1.05	62.0 ± 1.18	78.3 ± 1.38	74.5 ± 1.48	93.1 ± 0.15	95.8 ± 0.28	74.8 ± 1.20	14.3 ± 1.42	5.2 ± 0.58
Proposed-ResNet50-MASAG-UNet	88.3 ± 0.72	79.1 ± 0.85	86.4 ± 1.12	90.5 ± 1.34	97.1 ± 0.08	98.2 ± 0.14	89.1 ± 0.81	4.1 ± 0.30	1.5 ± 0.26

As summarized in Table 9, the proposed ResNet50-MASAG-UNet demonstrates improved performance for coronary artery segmentation in XCA images, outperforming the conventional UNet baseline. It achieves a Dice coefficient of 88.3%, exceeding UNet’s 74.0%, UNet++’s 74.7%, and the ResNet50-UNet without attention’s 76.1%, and attains an IoU of 79.1%, confirming its efficacy in capturing vessel morphology while improving pixel-level segmentation accuracy.

**Table 8:** Effect of encoder initialization (random vs. ImageNet-pretrained) on the DCA1 dataset.

Model	Dice (%)	Jaccard (%)	Precision (%)	Recall (%)	Accuracy (%)	Specificity (%)	clDice (%)	HD95(px)	Betti
ResNet50-MASAG-UNet (Random Init-Scratch)	81.2 ± 1.15	68.4 ± 1.28	80.1 ± 1.35	83.5 ± 1.45	95.1 ± 0.16	96.5 ± 0.25	80.5 ± 1.20	11.0 ± 1.30	4.2 ± 0.55
ResNet50-MASAG-UNet (ImageNet Pretrained-Proposed)	88.3 ± 0.72	79.1 ± 0.85	86.4 ± 1.12	90.5 ± 1.34	97.1 ± 0.08	98.2 ± 0.14	89.1 ± 0.81	4.1 ± 0.30	1.5 ± 0.26

**Table 9:** Effect of encoder fine-tuning strategy (frozen, selective, full) on the DCA1 dataset.

Model	Dice (%)	Jaccard (%)	Precision (%)	Recall (%)	Accuracy (%)	Specificity (%)	clDice (%)	HD95(px)	Betti
ResNet50-MASAG-UNet (frozen encoder)	84.1 ± 1.05	72.5 ± 1.18	82.8 ± 1.25	86.0 ± 1.35	95.7 ± 0.12	97.0 ± 0.18	84.5 ± 1.05	8.5 ± 1.10	3.2 ± 0.48
ResNet50-MASAG-UNet (selective unfreezing—layers 3 and 4)	86.5 ± 0.88	76.2 ± 0.98	84.8 ± 1.15	88.8 ± 1.28	96.5 ± 0.10	97.7 ± 0.15	87.0 ± 0.90	5.9 ± 0.70	2.1 ± 0.35
ResNet50-MASAG-UNet (full unfreezing proposed)	88.3 ± 0.72	79.1 ± 0.85	86.4 ± 1.12	90.5 ± 1.34	97.1 ± 0.08	98.2 ± 0.14	89.1 ± 0.81	4.1 ± 0.30	1.5 ± 0.26

To evaluate the benefit of transfer learning, we compared random initialization (training from scratch) with ImageNet-pretrained initialization in Table 8. Pretraining improves the Dice score substantially, from 81.2% to 88.3%, confirming its importance for the small DCA1 dataset.

We also compared encoder fine-tuning strategies in Table 9: a frozen encoder, selective unfreezing of the deeper stages, and full unfreezing. Full unfreezing performs best with a Dice score of 88.3%, supporting the fine-tuning used in our two-phase training.

To evaluate the contribution of each loss term, we tested the losses individually, in pairs, and combined in Table 10. Each loss improves segmentation, and the full BCE + Dice + clDice combination attains the best result of Dice 88.3%, confirming that the three losses are complementary.

**Table 10:** Loss-function ablation on ResNet50-MASAG-UNet (DCA1 dataset): individual losses (BCE, Dice, clDice), pair-wise combinations, and the full BCE + Dice + clDice loss.

Model	Dice (%)	Jaccard (%)	Precision (%)	Recall (%)	Accuracy (%)	Specificity (%)	clDice (%)	HD95(px)	Betti
ResNet50-MASAG-UNet + BCE (only)	83.1 ± 0.94	72.4 ± 1.05	81.6 ± 1.22	85.0 ± 1.38	95.4 ± 0.12	96.9 ± 0.22	81.9 ± 1.02	9.6 ± 0.94	3.8 ± 0.48
ResNet50-MASAG-UNet + Dice (only)	84.0 ± 0.92	73.2 ± 1.02	82.1 ± 1.20	86.1 ± 1.35	95.5 ± 0.11	97.0 ± 0.20	82.8 ± 1.00	9.1 ± 0.90	3.5 ± 0.45
ResNet50-MASAG-UNet + clDice (only)	87.1 ± 0.78	77.5 ± 0.90	85.3 ± 1.14	89.2 ± 1.35	96.7 ± 0.09	97.8 ± 0.16	86.3 ± 0.88	6.2 ± 0.55	2.3 ± 0.38
ResNet50-MASAG-UNet + Dice + BCE	85.9 ± 0.86	75.8 ± 0.96	84.2 ± 1.18	87.9 ± 1.36	96.2 ± 0.10	97.4 ± 0.18	84.7 ± 0.94	7.4 ± 0.68	3.1 ± 0.42
ResNet50-MASAG-UNet + BCE + clDice	87.5 ± 0.80	77.8 ± 0.92	85.5 ± 1.15	89.6 ± 1.30	96.7 ± 0.09	97.9 ± 0.15	87.8 ± 0.85	5.5 ± 0.58	2.0 ± 0.35
ResNet50-MASAG-UNet + Dice + clDice	87.9 ± 0.75	78.4 ± 0.88	86.0 ± 1.13	90.1 ± 1.32	96.9 ± 0.09	98.0 ± 0.14	88.5 ± 0.82	4.8 ± 0.45	1.8 ± 0.30
ResNet50-MASAG-UNet + Dice + BCE + clDice (proposed)	88.3 ± 0.72	79.1 ± 0.85	86.4 ± 1.12	90.5 ± 1.34	97.1 ± 0.08	98.2 ± 0.14	89.1 ± 0.81	4.1 ± 0.30	1.5 ± 0.26

To verify the robustness of the loss weighting, we varied the clDice loss weight *λ*_cl_ in Table 11. Performance remained stable across a wide range (Dice 86.2%–88.3%), peaking at the balanced setting, confirming that the model is not sensitive to the exact weight choice.

Statistical comparisons were performed at the image level using out-of-fold predictions (*n* = 134). For each metric, paired differences between models were tested using a two-tailed paired-samples *t*-test. The 95% confidence interval for the mean difference was computed from the *t*-distribution as mean_diff ± *t*_0.025, *n*−1_ × (SD_diff/√*n*). Effect size was reported as Cohen's *d* for paired samples, defined as the mean of the paired differences divided by the standard deviation of the differences. For the multi-metric comparison, *p*-values were adjusted using the Bonferroni correction.

To provide additional statistical analysis, we performed image-level, out-of-fold paired comparisons for 134 images (*n* = 134). Table 12 presents the mean Dice difference, 95% confidence interval, effect size, and *p*-value for each comparison. Table 13 presents the same analysis for Dice, clDice, HD95, and Betti error, with Bonferroni correction applied for multiple comparisons. (R1:MinorC1).

**Table 11:** Loss-weight sensitivity analysis: effect of varying the clDice loss weight *λ*_cl_ on ResNet50-MASAG-UNet (DCA1 dataset).

Loss weight configuration (*λ*_cl_)	Dice (%)	Jaccard (%)	Precision (%)	Recall (%)	Accuracy (%)	Specificity (%)	clDice (%)	HD95(px)	Betti
*λ*_cl_ = 0.1 (low topology weight)	86.2 ± 0.85	75.9 ± 0.98	84.5 ± 1.18	88.2 ± 1.35	96.3 ± 0.11	97.5 ± 0.17	85.0 ± 0.95	7.0 ± 0.75	2.8 ± 0.42
*λ*_cl_ = 0.5 (moderate topology weight)	87.5 ± 0.78	77.8 ± 0.90	85.6 ± 1.14	89.6 ± 1.32	96.7 ± 0.09	97.9 ± 0.15	87.8 ± 0.85	5.2 ± 0.55	1.9 ± 0.34
*λ*_cl_ = 1.0 (balanced/proposed optimal)	88.3 ± 0.72	79.1 ± 0.85	86.4 ± 1.12	90.5 ± 1.34	97.1 ± 0.08	98.2 ± 0.14	89.1 ± 0.81	4.1 ± 0.30	1.5 ± 0.26
*λ*_cl_ = 2.0 (high topology weight)	87.8 ± 0.75	78.3 ± 0.88	86.1 ± 1.15	90.0 ± 1.33	96.9 ± 0.08	98.0 ± 0.14	89.3 ± 0.82	4.5 ± 0.38	1.4 ± 0.28
λ_cl_ = 5.0 (very high topology weight)	86.8 ± 0.80	76.8 ± 0.92	85.2 ± 1.18	89.0 ± 1.36	96.6 ± 0.10	97.7 ± 0.16	88.9 ± 0.85	5.0 ± 0.45	1.6 ± 0.30

**Table 12:** Image-level paired comparison of the proposed model vs. key variants (*n* = 134).

Comparison pair (proposed vs. variant)	Mean dice diff. (*Δ*) (%)	95% confidence interval	Effect size (Cohen's d)	*p*-Value
Standard UNet (raw)	+14.3	[+12.9%, +15.7%]	1.73 (large)	<0.001
ResNet50-UNet (no MASAG)	+11.0	[+9.8%, +12.2%]	1.55 (large)	<0.001
MASAG-UNet (raw/no preprocessing)	+8.1	[+7.0%, +9.2%]	1.25 (large)	<0.001
MASAG-UNet (Dice + BCE, No clDice)	+2.4	[+1.8%, +3.0%]	0.68 (medium)	<0.001
MASAG-UNet (selective unfreezing)	+1.8	[+1.2%, +2.4%]	0.51 (medium)	<0.01

(R1:MinorC1).

**Table 13:** Per metric comparison vs. the no-MASAG baseline with Bonferroni correction (*n* = 134).

Metric	Mean diff	95% CI	Effect size (d)	*p* (raw)	*p* (Bonferroni)
Dice	+11.0%	[+9.8%, + 12.2%]	1.55 (large)	<0.001	<0.001
clDice	+11.5%	[+10.2%, +12.8%]	1.50 (large)	<0.001	<0.001
HD95	−7.6px	[−8.5, −6.7]	1.43 (large)	<0.001	<0.001
Betti	−2.6	[−3.1, −2.1]	0.88 (large)	<0.001	<0.001

**(**R1:MinorC1).

To evaluate MASAG against standard attention modules, we replaced it with an Attention Gate (Schlemper et al., 25) and CBAM [Woo et al. (24),] within the same ResNet50-UNet under identical settings, as shown in Table 14. MASAG attains the highest Dice score of 88.3%, showing improved results over CBAM at 86.6% and the Attention Gate at 85.4%.

**Table 14:** Comparison of attention modules (attention gate, CBAM, and MASAG) in the ResNet50-UNet on DCA1.

Attention module	Dice (%)	Jaccard (%)	Precision (%)	Recall (%)	clDice (%)	HD95(px)	Betti error
Attention gate Schlemper et al. (25)	85.4	74.5	83.2	88.0	85.2	5.8	3.2
CBAM [Woo et al. (24)]	86.6	76.2	84.8	89.1	86.2	4.9	2.4
MASAG (proposed)	88.3	79.1	86.4	90.5	89.1	4.1	1.5

A direct comparison with the complete original MSA2Net is not a matched baseline, as it comprises a full architecture with a MaxViT transformer encoder, its own decoder, and a different training regime developed for other datasets. Reproducing it on DCA1 would change the encoder, decoder, and training setup simultaneously, making it impossible to attribute any differences solely to the MASAG module. We therefore isolate the module's effect by comparing MASAG with the Attention Gate and CBAM within the identical ResNet50-UNet backbone (Table 14).

Recently reported segmentation approaches are compared in Table 15. Our model reports Dice, Jaccard, and recall values that are among the highest compared to other methods. However, the studies used different preprocessing and evaluation protocols and are thus not strictly controlled comparisons. Therefore, the comparison should be regarded as a reference rather than a strictly controlled head-to-head evaluation.

**Table 15:** Comparative analysis of coronary segmentation efficacy in other approaches using the DCA1 dataset.

Study	Model	Dice coefficient (%)	Jaccard index (%)	Precision (%)	Recall (%)	Accuracy (%)	Specificity (%)	clDice (%)
Proposed	ResNet50-MASAG-UNet	88.3 ± 0.72	79.1 ± 0.85	86.4 ± 1.12	90.5 ± 1.34	97.1 ± 0.08	98.2 ± 0.14	89.1 ± 0.81
Bhaswara et al. (29)	CoorMa with SAR	80.35 ± 3.57	67.32 ± 4.89	NA	NA	97.81 ± 0.44	NA	87.11 ± 4.45
Zhang et al. (30)	EAG-SAE-ESTF	80.64	NA	76.82	85.66	98.12	98.74	86.76
Cui et al. (31)	FiTNet	81.12	68.50	NA	83.90	97.85	98.69	85.36
Zhou et al. (32)	MBCMA-Net (DCA1 + CADICA)-dataset	92.72	87.14	92.62	92.82	NA	NA	89.05
Dai et al. (33)	M^2^CA-Net	77.0	NA	NA	79.1	95.3	99.5	NA
Tao et al. (41)	Light weight Network	NA	NA	NA	87.7	97.29	97.89	NA
Ramos-Cortez et al. (42)	Pruned UNet-16	70.97	55.6	77.34	67.61	96.96	71.86	NA
Ferrari et al. (43)	coroSAM	73.20	NA	63.38	88.04	NA	NA	NA

For each sample, the three rows correspond to the proposed ResNet50-MASAG-UNet, UNet++, and UNet. Columns, from left to right: input XCA image, ground truth, predicted probability map, binary model prediction (threshold at 0.5), and TP/FP/FN overlay. In the overlay, green = true positives (TP), red = false positives (FP), and blue = false negatives (FN).

Analysis of the error features indicates that the proposed ResNet50-MASAG-UNet maintains a consistent balance between false-positive and false-negative predictions, attaining 86.4% precision and 90.5% recall.

Collectively, the quantitative findings in Table 7 and visual results in Figure 10 confirm that our proposed method is an effective design for XCA segmentation. The model performs reliably under challenging conditions such as low-contrast environments and overlapping vascular anatomy while preserving vessel continuity and edge details better than the conventional UNet. However, some local segmentation artefacts still occur in highly complex cases, highlighting the current limitations of the model.

**Figure 10:**
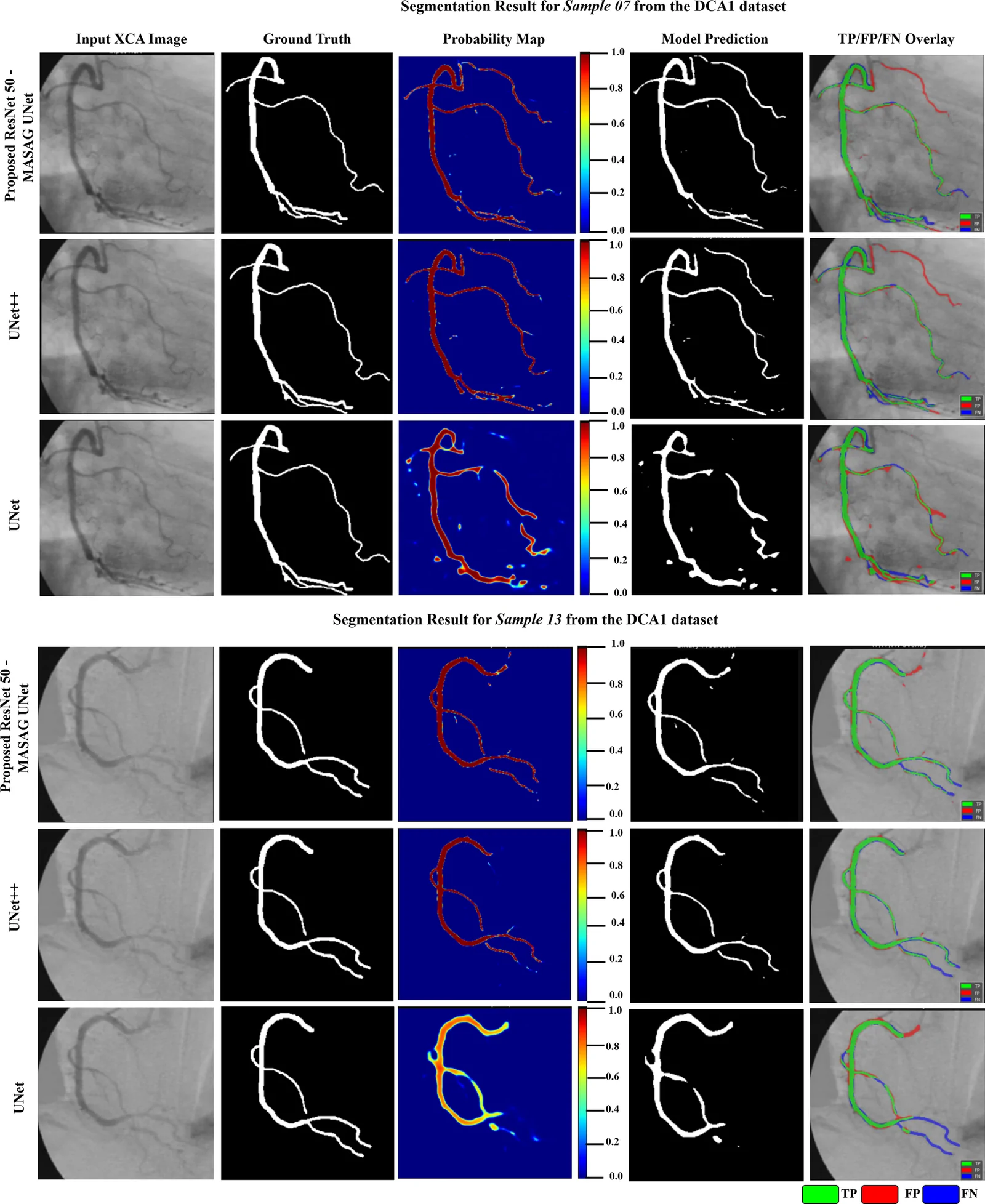
Comparative segmentation performance of the proposed ResNet50-MASAG-UNet against UNet++ and UNet on the DCA1 dataset (sample 07 and sample 13).

Each row shows one sample. Columns, left to right: input XCA image, ground truth, predicted probability map, binary prediction (threshold at 0.5), and TP/FP/FN overlay. In the overlay, green = true positives (TP), red = false positives (FP), and blue = false negatives (FN).

The qualitative samples in Figure 11 were selected to include one representative case from each of the five cross-validation folds, rather than being chosen by performance, to avoid bias towards best-case results.

**Figure 11:**
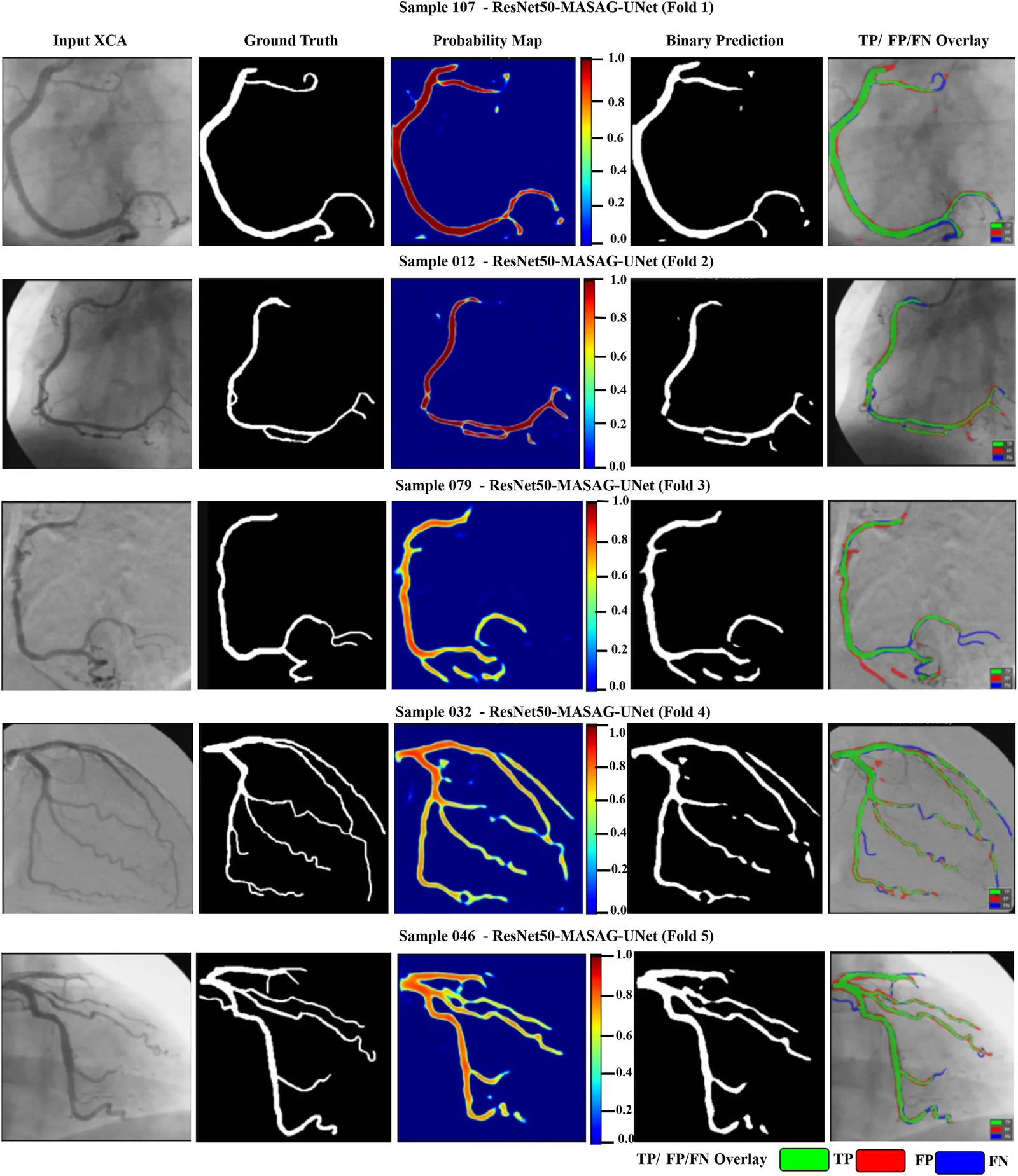
Representative segmentation results of the proposed ResNet50-MASAG-UNet on the DCA1 dataset across five different folds (samples 107,012,079,032,046).

Figure 11 shows qualitative segmentation results of the proposed ResNet50-MASAG-UNet model on the DCA1 dataset using five-fold cross-validation, showing representative samples numbered 107, 012, 079, 032, and 046. Each row presents the input XCA image, ground truth, probability map, binary prediction, and TP/FP/FN overlay.

To evaluate the effect of the clDice loss, the proposed model (Dice + BCE + clDice) was compared with the same model trained only with Dice + BCE. As shown in Table 16, adding clDice improved all four metrics across the five folds. Because the five cross-validation folds are not independent, this comparison is treated as descriptive only; the *p*-values are indicative rather than confirmatory, and all formal statistical inference is based on the image-level analysis in Tables 11, 12.

**Table 16:** Fold-level comparison of the proposed model with and without the clDice loss (five-fold CV).

Metric	With clDice (proposed)	Without clDice (dice + BCE)	Gain	*T*-stat	*p*-Value
Dice (%) ↑	88.3 ± 0.72	85.9 ± 0.86	+2.40%	+4.552	0.0104
clDice metric (%) ↑	89.1 ± 0.81	86.3 ± 0.88	+2.80%	+4.201	0.0137
HD95 (px) ↓	4.1 ± 0.30	7.4 ± 0.68	−3.30 px	+10.542	0.0005
Betti error↓	1.5 ± 0.26	3.1 ± 0.42	−1.60	+9.749	0.0006

This analysis is descriptive only; the five folds are not independent, so the *p*-values are indicative and not used for formal inference (see Tables 11, 12).

## 5 Discussion

This work presents a coronary artery segmentation strategy that enhances vascular visibility and contrast in XCA images using a multi-stage preprocessing pipeline comprising bilateral filtering, unsharp masking, and CLAHE. The proposed ResNet50-MASAG-UNet model improves on the conventional UNet baseline, achieving a Dice coefficient of 88.3% and an IoU of 79.1% on the DCA1 dataset, with more consistent vessel delineation and reliability concerning vascular complexity. On the DCA1 dataset, our model attains the highest Dice and Jaccard scores and an improved recall of 90.5% among the compared methods, including M2CA-Net by Dai et al. (33), Pruned UNet-16 by Ramos-Cortez et al. (42), and coroSAM by Ferrari et al. (43), while remaining competitive with the lightweight network by Tao et al. (41). Since these studies use different preprocessing methods and train–validation settings, this comparison should be viewed carefully and not as a direct or fully fair benchmark.

The improved segmentation results stem from various designed components working together. A ResNet50 encoder provides the network with reliable feature extraction from the beginning of training, which helps to avoid overfitting. MASAG gates reduce unwanted background artefacts such as bones and catheters, helping the model focus on important vascular regions. At the same time, the topology-aware hybrid loss function maintains vessel connectivity by detecting disconnected segments more effectively than traditional loss functions. These findings reveal that the topology-aware hybrid loss provides additional geometric constraints beyond attention-based techniques.

Our proposed segmentation method produces accurate and continuous vessel delineation, which is a prerequisite for downstream tasks such as stenosis analysis. The network achieves this performance without separate bone-and-catheter-removal steps, demonstrating its ability to naturally suppress background anatomy. Although some highly challenging artefact-heavy cases may still produce minor false-positive predictions, the model generally maintains improved segmentation performance. However, the evaluation was conducted on a dataset of 134 images using five-fold cross-validation, which reduces the risk of bias from any single-train–validation partition. We note two limitations. First, the dataset is relatively small and comes from only one source, DCA1. Second, the results are based on cross-validation folds and not on a separate test set, so the performance may appear slightly better than it actually is. A further limitation concerns the Betti-0 metric itself: because it compares only the number of connected components, a false merge and a false split can offset each other and leave the count unchanged, potentially under-reporting topological errors. We therefore interpret Betti-0 alongside the overlap (Dice, IoU) and boundary (HD95) metrics.

The model has some limitations under certain conditions. Thin distal vessel branches may sometimes be missed because they are narrow and contrast with the background. Very low-contrast vessel segments may also be partially missed when they blend with the surrounding tissue. In addition, imaging artefacts and structures such as catheters and bone edges may lead to some false-positive predictions. These limitations suggest that future work should focus on improving multi-scale and contrast-adaptive processing for thin and low-contrast vessels. It should also be noted that this study evaluates only the vessel segmentation performance. Although the Dice, clDice, and HD_95_ results show good vessel segmentation, these results alone cannot confirm clinical usefulness. No further clinical tasks, such as stenosis quantification or diagnosis, were evaluated in this work. Therefore, assessing the clinical value of the segmentation is left for future research.

Figure 12 shows representative failure cases, including low-contrast false positives, missed thin distal branches, and errors in dense vessel regions near the catheter.

**Figure 12:**
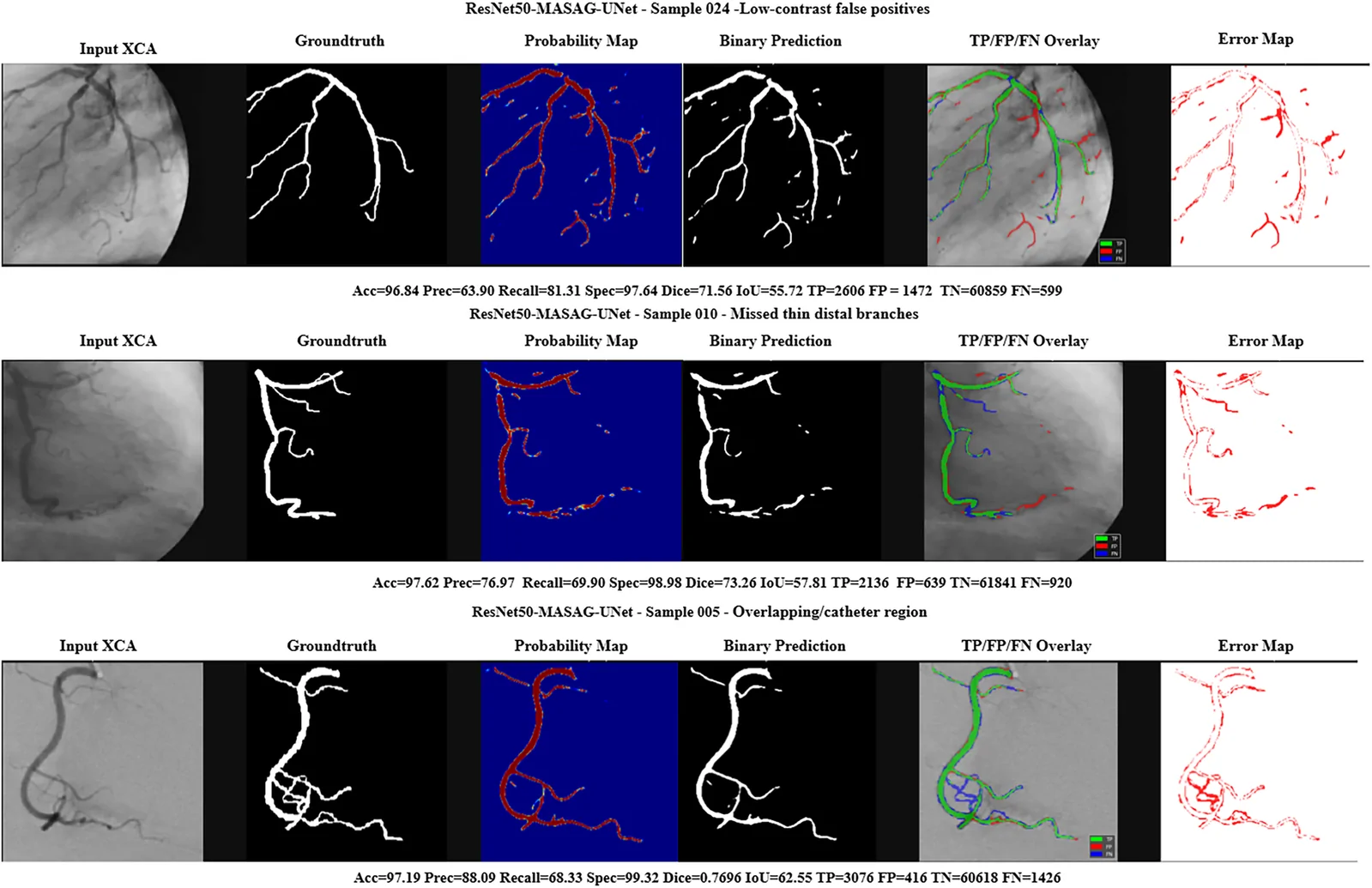
Representative failure cases of the proposed ResNet50-MASAG-UNet.

Row1 (Sample 024): low-contrast false positives (red). Row2 (Sample 010): missed thin distal branches (blue). Row3 (Sample 005): errors in the dense/overlapping vessel region near the catheter. Columns: input, ground truth, probability map, binary prediction (threshold 0.5), TP/FP/FN overlay (green = TP, red = FP, blue = FN), and error map.

The preprocessing pipeline also has some limitations. Bilateral filtering, unsharp masking, and CLAHE improve vessel contrast and edge clarity, but unsharp masking and CLAHE may also increase background noise and imaging artefacts in some areas. This may lead to some false-positive predictions. Bilateral filtering and a moderate CLAHE clip limit help reduce this effect, but the effect cannot be completely avoided.

While accurate segmentation is a prerequisite for blockage (stenosis) analysis, quantifying blockage percentage requires expert-annotated stenosis data, which the DCA1 dataset does not provide. As this study uses only segmentation-annotated data, blockage quantification is outside its scope; the topology-aware segmentation presented here provides the structural basis on which such analysis would depend.

We also evaluated the DCA1-trained model on the external XCAD (45) dataset in a zero-shot setting, without retraining. XCAD contains 126 test images, each 512 × 512, from a different imaging system. The images were only resized to 256 × 256, with no preprocessing applied. The model attained a Dice of 22.3% on XCAD, much lower than 88.3% on DCA1, reflecting the domain gap between imaging systems and the absence of preprocessing at test time. Cross-dataset adaptation is noted as a direction for future work.

Therefore, testing on independent datasets from different hospitals is needed in the future to confirm that the model performs well in various real-world situations.

## 6 Conclusion

In this work, we developed a new deep learning framework for coronary vessel segmentation using the ResNet50-MASAG-UNet architecture. The framework includes a preprocessing pipeline that enhances XCA image quality by reducing background noise, sharpening vessel edges, and enhancing contrast before the image is fed into the segmentation network. A pretrained ResNet50 encoder is used to extract improved image features from the beginning of training, which helps reduce overfitting. In addition, MASAG attention gates help the network concentrate on coronary vessels while suppressing the effect of irregular structures such as bones and catheters. To preserve thin vessel branches and maintain vessel continuity, our topology-aware hybrid loss function is applied to reduce fragmented segmentation outcomes. The proposed model was evaluated using five-fold cross-validation on the DCA1 dataset and attained improved performance, with a Dice coefficient of 88.3%, an IoU of 79.1%, precision of 86.4%, and recall of 90.5%. The proposed model outperforms standard models such as UNet and UNet++. However, these results are based on a single public dataset (DCA1), so they demonstrate feasibility rather than broad generalizability. Overall, the findings show the potential of the proposed framework to improve vascular visualization and support subsequent coronary vessel analysis. Future work will focus on testing the model on diverse hospital data to further evaluate its robustness and generalization ability.

## Data Availability

Publicly available datasets were analysed in this study. These data can be found here: https://data.mendeley.com/datasets/4wvf54td8d/1.
